# BMP-signaling in the intestinal epithelium drives a critical feedback loop to restrain IL-13-driven tuft cell hyperplasia

**DOI:** 10.1126/sciimmunol.abl6543

**Published:** 2022-05-13

**Authors:** Håvard T. Lindholm, Naveen Parmar, Claire Drurey, Marta Campillo Poveda, Pia Vornewald, Jenny Ostrop, Alberto Díez-Sanchez, Rick M. Maizels, Menno J. Oudhoff

**Affiliations:** 1 CEMIR - Centre of Molecular Inflammation Research, Department of Clinical and Molecular Medicine, NTNU - Norwegian University of Science and Technology, 7491 Trondheim, Norway; 2 Wellcome Centre for Integrative Parasitology, Institute of Infection, Immunology and Inflammation, University of Glasgow, G12 8TA Glasgow, United Kingdom

## Abstract

The intestinal tract is a common site for different types of infections including viruses, bacteria, and helminths, each requiring specific modes of immune defense. The intestinal epithelium has a pivotal role in both immune initiation and effector stages, which are coordinated by immune-type specific cytokines such as IFNγ, IL-13 and IL-22. Here, we study intestinal epithelial immune responses using organoid image analysis based on a convolutional neural network, transcriptomic analysis, and *in vivo* infection models. We find that IL-22 and IL-13 both induce genes associated with goblet cells, but their phenotypes are dichotomous. Moreover, only IL-13 driven goblet cells are associated with classical NOTCH signaling. We further show that IL-13 induces BMP signaling, which acts in a negative feedback loop in immune type 2 driven tuft cell hyperplasia. This is associated with inhibiting *Sox4* expression to putatively limit the tuft progenitor population. Blocking BMP signaling with the ALK2 inhibitor DMH1 interrupts the feedback loop, resulting in greater tuft cell numbers both in *vitro* and in vivo after infection with *Nippostrongylus brasiliensis.* Taken together, these novel aspects of cytokine effector responses reveal an unexpected and critical role for BMP signaling in type 2 immunity, which can be exploited to tailor epithelial immune responses.

## Introduction

Gut infections remain a common threat for patients and are an immense burden on health systems worldwide (1). Resistance to intestinal pathogens relies on the capacity of the immune system to mount an appropriate response. For example, one requires a different type of response to intracellular viruses compared to extracellular pathogens including bacteria or parasites. Cytokines are key participants in polarizing the immune response by altering the cellular composition and state. Innate lymphoid cells (ILCs) are tissue resident immune cells which are early responders to infections and create a local cytokine environment. ILCs are classically divided into three groups and secrete IFNγ (group 1), IL-13 (group 2), and IL-22 (group 3), and which ILC subtype is dominant depends on the pathogenic challenge (2).

In addition to defining the immune landscape, cytokines directly affect intestinal epithelial cells (IECs) to drive immune-type specific responses (3). The intestinal epithelium consists of a single layer of cells and is responsible both for taking up nutrients as well as providing a protective barrier. One of the hallmarks of IECs is their rapid turnover (3-5 days), which allows for prompt cellular responses, for example, to expand goblet cells which can produce protective mucus. This plasticity of IECs makes them particularly well-suited to defend against pathogens (4).

In addition to responding to immune cues, the epithelium can also be involved in tailoring immune responses. For example, tuft cells, which are important for defending against parasitic helminths, are the main source of IL-25 to control ILC2 populations both in homeostasis and upon helminth infection (5–7). As tuft cells rapidly expand upon exposure to type 2 cytokines, they exemplify both how the intestinal epithelium changes upon an immune response and how it can partake in shaping it. Of note, tuft cells are not only important in immunity to helminth infections; activation of tuft cells by succinate is protective in a murine model of colitis, and reduced tuft cell numbers are found in patients with Crohn’s disease that have more severe inflammation (8). Thus, tuft cells have been gaining interest as important regulators of intestinal diseases (9), and the discovery of regulatory mechanisms could have clinical ramifications.

Intestinal organoids are self-organizing structures that are useful to study epithelial (stem) cell biology (10). They are particularly instructive to identify epithelial-intrinsic responses as they lack any other cell type normally present *in vivo*, such as fibroblasts. This also means their culture medium requires addition of growth factors normally supplied by fibroblasts, such as those that target pathways such as WNT and BMP. In addition, organoid cultures come with challenges as most metrics cannot capture the complexity of these large multicellular structures. To capture this complexity, a study used single cell RNA sequencing (scRNA-seq) to systemically compare single cell responses to cytokines of the three immune environments (3). However, there are still many unclear aspects to how cytokines induce epithelial responses, especially mechanistically. Here we combine quantitative imaging with bulk RNA-seq to define how cytokines intercept developmental pathways to instruct epithelial differentiation and maturation. Most prominently, we identify a feedback loop by which IL-13 induced tuft cell hyperplasia is self-limiting in a BMP-dependent manner, and confirm this in a murine helminth infection model using *N. Brasiliensis*.

## Results

### IFNγ, IL-13, and IL-22 uniquely affect organoid growth and morphology

**Figure 1:**
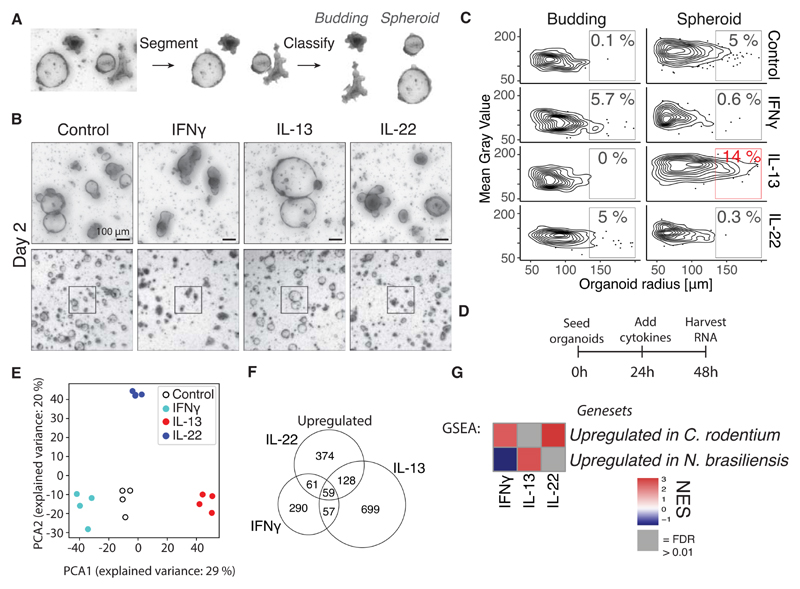
Cytokines modify development of small intestinal epithelium in a cytokine-specific manner and correspond to *in vivo* infection models. **A**, Image segmentation and classification of organoid images. **B**, Bright field images of small intestinal organoids treated with 10 ng/mL cytokine since the day of splitting. Images are projection of Z-stack. **C**, Distribution of gray value (higher value is whiter) and area of all organoids in same experiment as B. Percentages are relative to total number of organoids in that treatment. Plot shows distribution of in total 2,310 organoids, representative result of 5 mice. **D**, Time points for RNAseq experiment from small intestinal organoids treated with 10 ng/mL cytokine. **E**, PCA plot of log2(TPM + 1) values determined with RNAseq as shown in D. Each circle is one biological replicate. **F**, Number of up-regulated significant genes from same RNAseq experiment as in D (p<0.05 and log2fc>1). **G**, GSEA using genesets consisting of the 300 most significantly up-regulated genes from intestinal epithelium from mice infected with *N. brasiliensis* or *C. rodentium.* These genesets are compared to RNAseq of intestinal organoids stimulated with indicated cytokine compared to control. See supplementary file 1 for genesets. NES = normalised enrichment score.

Intestinal organoids grow as a morphologically heterogeneous population, where a fraction of organoids grow as immature “spheroids” and the rest form mature “budding” organoids. *Lgr5+* stem cells and Paneth cells are found in the buds in budding organoids (mimicking crypts) while spheroids consist of less differentiated, proliferating cells (11). Building on our recent work on organoid segmentation (12), we here developed an automated image analysis pipeline that segments organoid objects from the background and subsequently classifies them into “spheroid” or “budding” categories based on a convolutional neural network (Fig. 1A). This includes an optional manual correction step for organoids that were difficult to automatically segment or classify (Fig. S1A). Comparing manually verified with automatically classified and segmented images had a good correlation in analysis of >20,000 organoids (Fig. S1B). Expectedly, we find that in time the number of spheroids decrease and appear darker in appearance, confirming that our systematic approach captures what is observed visually (Fig. S1C,D,E).

To mimic different types of immune responses, we treated organoids with key cytokines IFNγ, IL-13, or IL-22. As was previously found (13, 14), long term IL-22 or IFNγ treatment ultimately leads to organoid disintegration (Fig. S1F). At an earlier time point (day 2), this is characterized by a darker appearance, reduced percentage of spheroids, and an increased percentage of budding organoids (Fig. 1B,C and S1G). In contrast, a population of IL-13 treated organoids form large spheroids at day 2 (Fig. 1C and S1G).

Tuft cells are a specific epithelial cell type involved in type 2 immunity, and induced by IL-13 (6, 7). Tuft cells in organoids share the characteristic shape and F-actin brush with their *in vivo* counterparts (Fig. S2A) (15). We next combined our classification setup with confocal imaging to automatically quantify tuft cells in organoid subtypes. This configuration allows for classification and counting of tuft cells in hundreds of organoids and supports both automatic estimates of tuft cell number and a more accurate manually curated count (Fig. S2B). Indeed, we find that tuft cells primarily appear in budding organoids both in control and IL-13-treated conditions (Fig. S2C,D,E). Together, these data show that classification of organoids combined with confocal imaging provides a tractable measure of epithelial cellular responses.

### IFNγ, IL-13, and IL-22 uniquely affect RNA expression in organoids in a manner aligned to *in vivo* infection profiles

To assess in detail how IFNγ, IL-13, and IL-22 affect intestinal epithelial cells we performed bulk RNA-seq on organoids treated with indicated cytokines for 24 hours compared to untreated controls (Fig. 1D). The different cytokines showed up-regulation of unique genes that grouped separately in a PCA plot, and each cytokine induced different GO-terms, highlighting the different effector responses required to each type of immune response (Fig. 1E,F, S3A-D).

To investigate to what degree cytokines control the epithelial response *in vivo*, we performed RNA-seq on intestinal epithelium from mice infected with *Nippostrongylus brasiliensis* or *Citrobacter rodentium,* which are classical intestinal infection models for a parasitic and extracellular bacterial infection respectively (Fig. S4A). Intestinal epithelium infected with *N. brasiliensis* did show the expected induction of tuft cells (Fig. S4C,D,E) and had up-regulation of GO terms associated with wound healing (Fig. S4F). Intestinal epithelium infected with *C. rodentium* had up-regulation of GO-terms associated with inflammatory response (Fig. S4G). We found that epithelium from duodenum isolated from *N. brasiliensis* infected mice was similar to IL-13 treated-organoids (Fig. 1G). In contrast, colonic epithelium isolated from *C. rodentium* infected mice aligned with IL-22- and IFNγ-treated organoids using gene set enrichment analysis (GSEA) (Fig. 1G). A caveat to the analysis of *C. rodentium* analysis is that this epithelium is isolated from colon and not small intestine. The correlation with IL-13 induced genes in *N. brasiliensis* infected epithelium and IL-22 induced genes in *C. rodentium* infected intestinal epithelium aligns with the standard model of the involvement of these cytokines in specific immune responses and indicates that treating intestinal organoids with cytokines provide a relevant model to, in part, mimic *in vivo* epithelial responses.

### IL-13 and IL-22 induce different gene signatures in goblet cells

**Figure 2:**
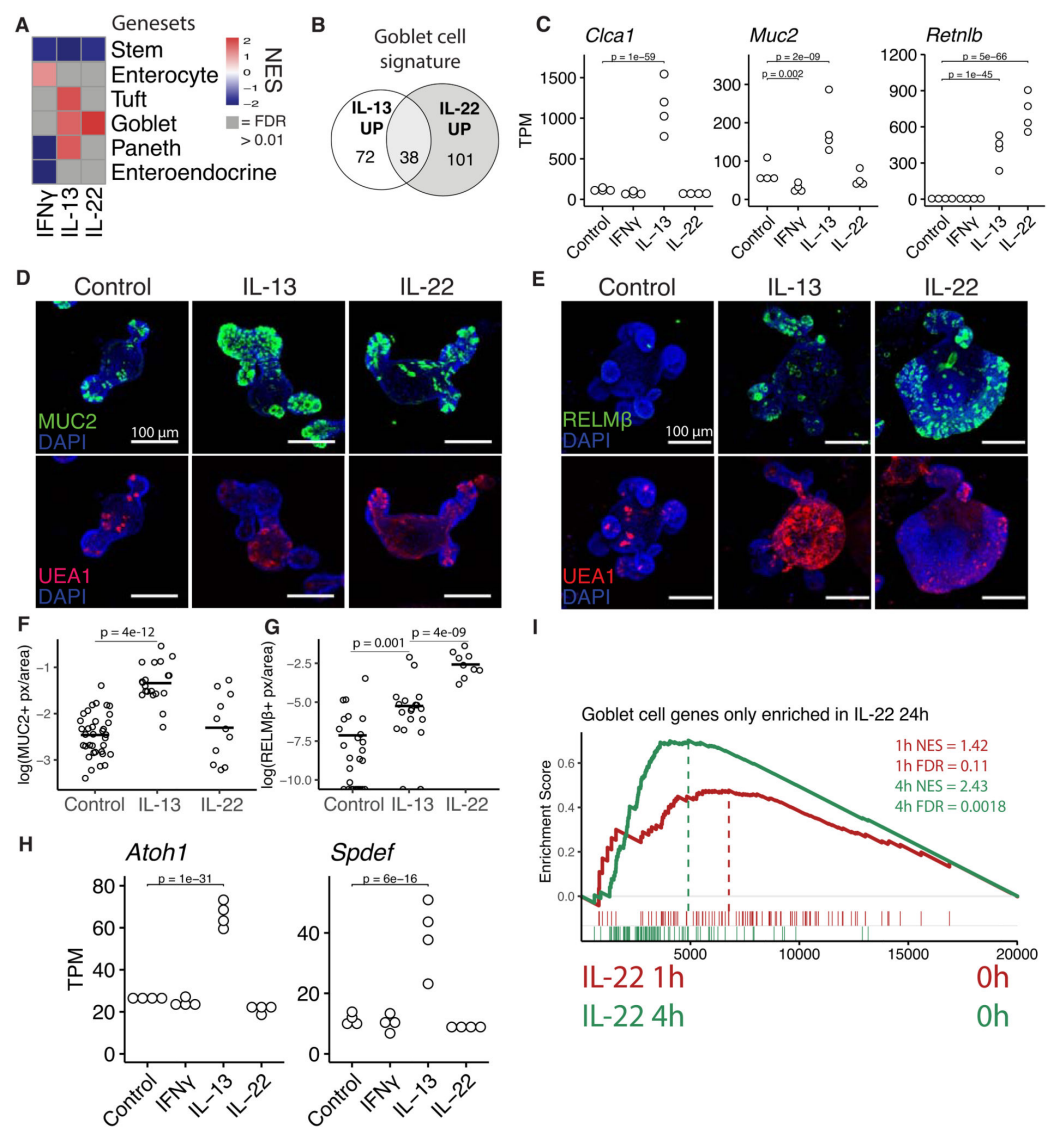
IL-13 and IL-22 induce different subsets of goblet cell genes. **A**, Heatmap of NES values from GSEA analysis of gene sets representing different cell types compared to RNAseq data from organoids treated for 24 hours with cytokines, see supplementary file 1 for gene sets. **B**, Distribution of how the goblet cell gene set from plate based scRNAseq from Haber et al is changed upon 24 hour IL-13 and IL-22 treatment in organoids. Up is defined as log2fc>0.5 and p-adj<0.05. **C**, Gene expression from intestinal organoids treated for 24 hours with indicated cytokine. Statistics calculated with DESeq2, details in methods. **D, E**, Confocal staining of MUC2 and UEA1 2 days after splitting (D) and RELMβ and UEA1 at 3 days after splitting (E) in small intestinal organoids. Representative images of three mice. 5 ng/mL of IL-22 and 10 ng/mL of IL-13 was used. **F,G**, Quantification of images acquired as in D and E. Pixels are defined as positive above a set threshold. Each circle represents one organoid and statistics calculated with unpaired two tailed T-test. **H**, See C. **I**, GSEA of IL-22 specific goblet cell genes from B compared to RNAseq data from organoids stimulated with IL-22 for 1h and 4h. All images are projections of Z-stacks.

To test the effect of IFNγ, IL-13 and IL-22 on cell lineage differentiation, we used cell-type specific gene signatures acquired through scRNAseq (16). GSEA revealed that these cytokines broadly affect intestinal cell lineage differentiation in an expected manner (Fig. 2A). For example, giving IL-13 to organoids induced a tuft cell signature, supported by tuft cell staining *in vitro* (Fig. S2C) and *in vivo* upon *N. brasiliensis* infection (Fig. S4C,D,E). In addition, both IL-13 and IL-22 induced a goblet-cell associated gene signature (Fig. 2A). However, close examination showed that each cytokine induced a different set of goblet-cell genes with relatively little overlap both after 24 and 72 hours of cytokine stimulation (Fig. 2B, S5A), see supplementary file 2 for complete gene lists. This is exemplified by goblet cell markers *Muc2* and *Clca1* being specifically induced by IL-13 whereas another goblet cell marker, RELMβ (*Retnlb*), was induced more strongly by IL-22 (Fig. 2C, S5B). Even though not all mucins are goblet cell specific (17), we found mucin genes to have different expression patterns between IL-13 and IL-22 treatment (Fig. S5C). Confocal staining confirms that MUC2 was induced by IL-13 (Fig. 2D,F), and that RELMβ was more strongly induced by IL-22 (Fig. 2E,G). We noted that IL-22 induced (RELMβ+) goblet cells also looked different from those induced by IL-13 and often lacked large granule-like structures (Fig. 2E). To determine if these cells where positive for *Muc2* by mRNA, we combined staining of *Muc2* by RNAscope with MUC2 protein staining (Fig. S5D). We found that IL-22 led to the expansion of *Muc2*-high cells with patterns mimicking (RELMβ+) staining (Fig. S5D). These cells also had low MUC2 protein levels. Canonical differentiation of goblet cells occurs through inhibition of NOTCH and relies on transcription factors ATOH1 and SPDEF (18). Indeed, IL-13 induced *Atoh1* and *Spdef*, however, IL-22 did not, indicating that IL-22 induces goblet cell genes in a non-typical manner (Fig. 2H, S5E). It is not clear from these results what mechanism leads to induction of IL-22 dependent goblet cell, but RNAseq of early time points after IL-22 stimulation show that *Relmβ* is induced after just a few hours and GSEA of IL-22 specific goblet cell genes show significant enrichment after just 4 hours (Fig. S5F, 2I). These results indicate that IL-22 specific goblet cell genes are relative direct targets of IL-22. Furthermore, GO-term analysis of the goblet cell genes uniquely induced by IL-13 and IL-22 revealed different terms, for example, “response to endoplasmatic stress” was the top GO term associated with goblet cell genes induced by IL-22 (Fig. S5G,H). Taken together, we propose that cytokine-driven goblet cell responses are linked to their function. IL-13 would primarily induce mucus to aid in parasite clearance whereas IL-22 responses are characterized by induction of antimicrobials to kill extracellular pathogens. More specifically, IL-13 leads to a quantitative increase in relative goblet cell numbers, whereas IL-22 treatment leads to a change in qualitative goblet cellular state including the induction of high levels of RELMβ.

### BMP signaling is associated with IL-13 and limits tuft cell differentiation *in vitro*

**Figure 3:**
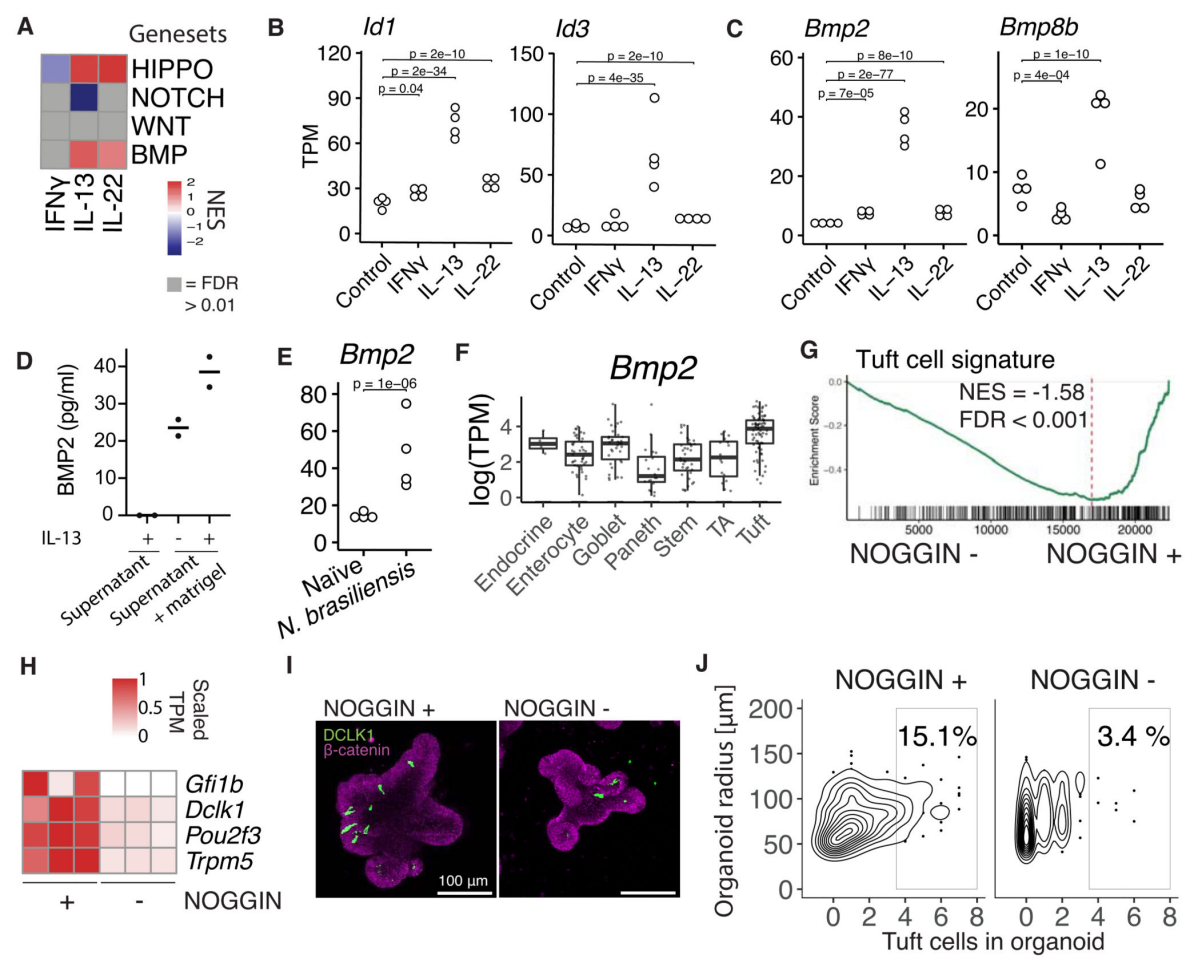
IL-13 induce BMP2 in intestinal epithelium. **A**, GSEA analysis of gene sets representing developmental pathways important in IEC development of bulk RNAseq data from organoids treated with 10 ng/mL cytokine for 24 hours. Gene set sources: HIPPO: Genes up in artifical YAP induction and down-regulated in YAP-KO. WNT: Genes up in organoids treated with the GSK3 inhibitor CHIR, NOTCH: Genes up in organoids from ATOH1 KO epithelium compared to wt and BMP: Genes up in organoid treated with BMP4 compared to control. Gene sets and references in supplementary file 1. **B,C**, Gene expression in small intestinal organoids treated with cytokines for 24 hours determined with RNAseq. Each dot is one biological replicate. **D**, Concentration of BMP2 in supernatant or supernatant+matrigel (without organoids) of organoids stimulated with IL-13 determined with ELISA. Each dot is one biological replicate. Representative of two experiments. **E**, Gene expression in epithelium from duodenum extracted from mice infected with *N. brasiliensis* determined with RNAseq. Each dot is one biological replicate. **F**, Plate based scRNAseq expression data from Haber et al in small intestinal epithelium. TA = Transit amplifying. **G**, GSEA of tuft cell gene set on RNAseq of small intestinal organoids grown in the presence of NOGGIN or not for 5 days since splitting. **H**, Tuft cell marker genes from same RNAseq data as G. **I**, DCLK1 confocal staining of small intestinal organoids grown in the presence of NOGGIN or not for 72 hours since splitting. Images are projections of Z-stacks. **J**, Quantitation of experiment in I. Representative of two experiments. Plot shows a total of 462 organoids. TPM = transcripts per million, NES = normalized enrichment score.

We were interested in how cytokines control mechanisms that define cell fate in the intestinal epithelium. Intestinal epithelium relies on NOTCH-, WNT-, BMP- and HIPPO-signaling to maintain homeostatic differentiation of cell lineages. We hypothesized that cytokines may use these developmental pathways in directing epithelial cell differentiation. We generated gene sets for these different pathways from published transcriptome datasets (19–22). GSEA analysis revealed that cytokines, and in particular IL-13 and IL-22, alter transcription of genes normally associated with HIPPO, NOTCH, and BMP pathways (Fig. 3A).

We were somewhat surprised to find a strong enrichment of BMP signaling upon IL-13 treatment. BMP members are traditionally expressed by mesenchymal cells, so it is unclear how IL-13 may induce BMP signaling. Nonetheless, established BMP target genes *Id1* and *Id3* (20), are upregulated specifically after IL-13 treatment (Fig. 3B). Next, we assessed the expression pattern of the TGF-β family members and surprisingly found that IL-13 robustly induced *Bmp2* and *Bmp8b* but not any other members (Fig. 3C, S6A). The change in the Bmp2 gene also was reflected with an increase of BMP2 protein secreted by organoids stimulated with IL-13 (Fig. 3D). In support, there was also an increase of *Bmp2* during a *N. brasiliensis* infection (Fig. 3E). Interestingly, Haber et al. lists *Bmp2* as a bona fide tuft cell marker in their gene sets from plate-based scRNAseq of small intestinal epithelium (Fig. 3F) (16). The connection between tuft cells and IL-13 signaling is further highlighted by the fact that tuft cells specifically expressed high levels of *IL13ra1* as noted by Haber et al., whereas *IL4ra* or other cytokine receptors did not have such skewed cell-type specific receptor expression (Fig. S6B-D). In addition, tuft cells have increased pSTAT6 levels compared to other lineages (23). To test whether BMP signaling affects tuft cell differentiation, we compared organoids grown in normal EGF, NOGGIN, and RSPO (ENR) media with organoids grown without the presence of the BMP antagonist NOGGIN (ER media). Thus, taking away the obstructing factor for BMP activation. Organoids grown with NOGGIN in the media showed enrichment for a tuft cell signature and had higher expression of established tuft cell markers (Fig. 3G,H). Furthermore, antibody staining of the tuft cell marker DCLK1 revealed higher levels of tuft cells in organoids grown in the presence of NOGGIN (Fig. 3I,J).

### BMP signaling acts as a feedback loop to limit tuft cell expansion

**Figure 4:**
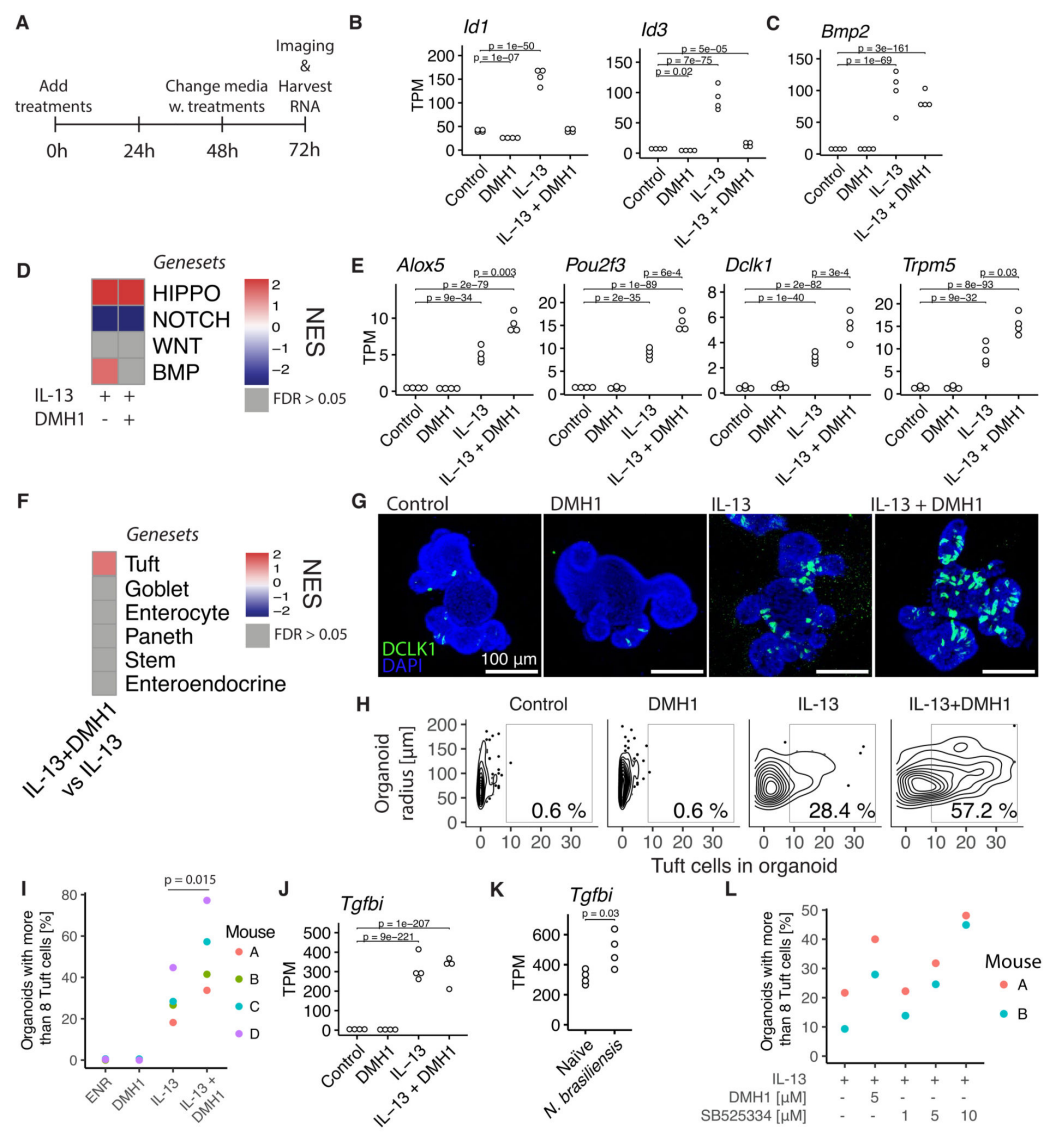
BMP signaling restricts IL-13-induced tuft cell expansion. **A**, Time points since seeding for RNAseq experiment from small intestinal organoids treated with 10 ng/mL cytokine and 5 μM DMH1 for 72 hours. **B,C**, Gene expression determined with RNAseq as described in A. Each circle is one biological replicate and statistics calculated with DESeq2, see methods. **D**, Heatmap of NES values from GSEA of gene sets representing signaling pathways in indicated treatments vs control condition. See supplementary file 1 for exact gene sets. **E**, see B. **F**, Heatmap of NES values from GSEA of gene sets representing cell types from Haber et al. in IL-13 + DMH1 treated organoids vs IL-13 organoids. See supplementary file 1 for exact gene sets. **G**, Confocal staining of DCLK1 in small intestinal organoids treated with indicated treatments for 72 hours. Images are projections of Z-stacks. **H**, Manual quantification of same experiment as G, plot represents a total of 566 organoids. **I**, Percentage determined as in H where each circle is a biological replicate from independent experiments. p-value determined with a paired T-test. **J**, See B. **K**, Gene expression in intestinal epithelium extracted from mice infected with *N. brasiliensis* determined with RNAseq. Each circle is one biological replicate and statistics calculated with DESeq2, see methods. **L**, Percentage of organoids with more than 8 tuft cells. Determined in similar fashion as in previous figures (see G,H and I) except that this is automatic tuft cell counts. Each circle is one biological replicate. Representative of two experiments. DMH1 inhibits ALK2 and SB525334 inhibits ALK5. TPM = transcripts per million, NES = Normalized Enrichment Score.

Tuft cells are crucial mediators of parasitic immunity. In a feed-forward loop, tuft cells amplify ILC2s by expressing IL-25, and ILC2s, in turn, express IL-13 to expand tuft cells (5–7). Our findings so far may suggest a novel mechanism that IL-13 treatment induces an epithelial-intrinsic feedback loop mediated by BMP signaling. To further investigate this, we tested the ALK2 (BMP type I receptor) inhibitor dorsomorphin homolog 1 (DMH1) in combination with IL-13 (24) (Fig. 4A). We found that DMH1 completely blocked the induction of canonical BMP target genes *Id1* and *Id3* (Fig. 4B), but did not affect IL-13-induced *Bmp2* expression (Fig. 4C). Furthermore, the overall enrichment of BMP signaling target genes by IL-13 is blocked by DMH1, without modulating the effect of IL-13 on HIPPO or NOTCH target genes (Fig. 4D). Although DMH1 in itself did not change expression of tuft cell-associated genes such as *Dclk1, Pou2f3, Trpm5,* and *Alox5,* it did increase the expression of these genes when combined with IL-13 (Fig. 4E). The tuft cell marker gene *Il25* could not be detected in our RNAseq, but we did see an increase in *Il25* in IL-13+DMH1 treatmed compared to DMH1 alone when we tested this separately by qPCR (Fig. S7A). In support, the tuft cell gene signature was enriched in IL-13 + DMH1 compared to IL-13 by GSEA analysis while no other cell type gene signature was altered (Fig. 4F). Confocal staining confirmed the specific enrichment of tuft cells upon combination treatment of IL-13 and DMH1 (Fig. 4G,H,I). In addition, we noted that IL-13 also induced expression of the TGF-β induced gene *Tgfbi,* independently of ALK2 (Fig. 4J), and this also occurred after *N. brasiliensis* infection (Fig. 4K). BMP signaling is complex and is mediated by multiple receptors. For example, BMP2 can activate both SMAD1/5/8 (BMP) and SMAD2/3 (TGF-β) signaling (25). Therefore, we decided to also test SB525334, an inhibitor for the TGF-β type I receptor ALK5. Just as seen with DMH1, we see an increase in IL-13 induced tuft cells in organoids treated with SB525334 in a dose-dependent manner (Fig. 4L, S7B,C). Together, this supports a model in which activation of BMP and/or TGF-β signaling limits IL-13-induced tuft cell differentiation, thus, providing a feedback loop to limit tuft cell expansion during immune responses.

### IL-13 activates BMP signaling in stem cells

**Figure 5:**
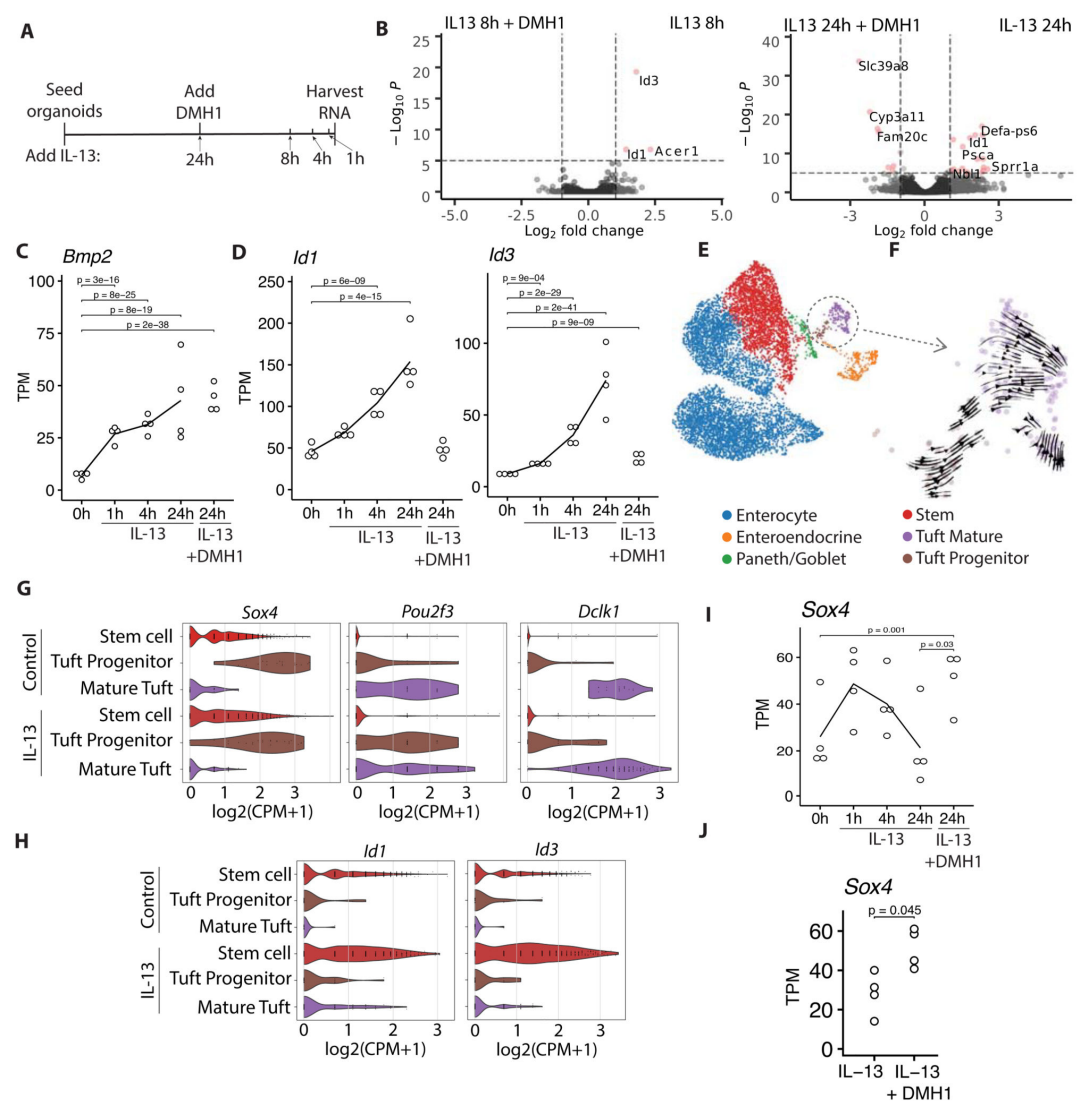
IL-13 activates BMP signaling in stem cells, skewing them away from tuft cell progenitors. **A**, Time points for RNAseq experiment from small intestinal organoids treated with 10 ng/mL cytokine at indicated timepoints and with and without 5 μM DMH1 for 24 hours. **B**, Volcano plots comparing indicated treatments from experiment described in A. **C, D**, Gene expression of experiment described in A. Each circle is one biological replicate, statistics calculated with DESeq2, see methods. **E**, scRNAseq data of small intestinal organoids treated with IL-13 and untreated from Biton et al (3). **F**, RNA velocity analysis of tuft cell populations from scRNAseq data presented in E. **G, H**, Violin plots of gene expression in tuft cells from scRNAseq data presented in E. **I**, See C. **J**, Gene expression of small intestinal organoids treated with indicated treatment for 72 hours. Each circle is one biological replicate, statistics calculated with DESeq2, see methods. TPM = transcripts per million, CPM = Counts per million.

To gain further insight into what role BMP signaling plays in the effect of IL-13 on the intestinal epithelium we stimulated small intestinal organoids with IL-13 for 1, 4, 8 and 24 hours with and without DMH1 (Fig. 5A). We found that IL-13 itself rapidly affects the transcriptome with 91 significantly changed genes after 1 hour compared to untreated, and 770 genes after 8 hours (p<0.01). In contrast, comparing DMH1+IL-13 *vs.* IL-13 we found a slower response with only 13 significantly changed genes after 8 hours, but 188 genes after 24 hours (p<0.01) (Fig. 5B). This suggests that BMP target genes are not early response genes upon IL-13 treatment. In accordance, we find that *Bmp2* and *Bmp8b* is rapidly induced after 1 hour (Fig. 5C, S8A), whereas maximal upregulation of canonical BMP target genes *Id1* and *Id3* and TGF-β target gene *Tgfbi* occurs only after 24 hours (Fig. 5D, S8B). Together, we propose that IL-13 mediated BMP signaling is mediated by rapid induction of BMP family members such as *Bmp2* and *Bmp8b* to subsequently activate BMP receptors.

We have found that BMP signaling limits IL-13-mediated tuft cell expansion (Fig. 4 and 5). To determine the cellular sequence of events, we downloaded and re-analyzed a scRNAseq data set from small intestinal organoids that were treated with IL-13 (Fig. 5E) (3). We split the tuft cell population in two, and by RNA velocity analysis we found that there is a tuft progenitor (with closer proximity to the stem cell population) and a mature tuft cell population (Fig. 5F). The mature tuft cells have a high expression of *Dclk1, Pou2f3* is expressed in both populations, and *Sox4* is specific for tuft cell progenitors (Fig. 5G). The trancription factor *Sox4* has previously been found to be important in tuft cell development (26). Although *Sox4* has been associated with the intestinal stem cell signature (27), in this dataset it is more highly correlated with progenitor tuft cells (Fig. 5G). Qi et al. found that mice with an inducible epithelial specific knock out of *Bmpr1a* (ALK3) had up-regulation of *Sox4* expression in *Lgr5*+ stem cells and that *Sox4* is down-regulated in *Lgr5*+ stem cells from intestinal organoids stimulated with BMP4 (20), indicating that BMP signaling might actively regulate the amount of *Sox4* positive tuft cell progenitors. To investigate where BMP signaling affected tuft cell development we plotted *Id1* and *Id3* expression and found them to be specifically induced in stem cells by IL-13 (Fig. 5H). Furthermore, *Sox4* is rapidly induced by IL-13 before it returns to homeostasis levels after 24 hours and this down-regulation is not seen in 24 hours of IL-13 stimulation with DMH1 (Fig. 5I). This result is supported by our 72 hour stimulation data where we see an up-regulation of *Sox4* in IL-13 + DMH1 compared to IL-13 (Fig. 5J). We were not able to associate receptor (*Acvr1*) expression to stem cells specifically (Fig. S8C), so how stem-cell enriched responses occur is unclear. In summary, these data indicate a model where IL-13 induces differentiation of stem cells into tuft cell progenitors positive for *Sox4.* At the same time, IL-13 induces production of BMP signaling molecules, which act on stem cells and inhibits them from developing into *Sox4* positive tuft cell progenitors.

### BMP signaling restricts *N. brasiliensis* induced tuft cell expansion in *vivo*

**Figure 6:**
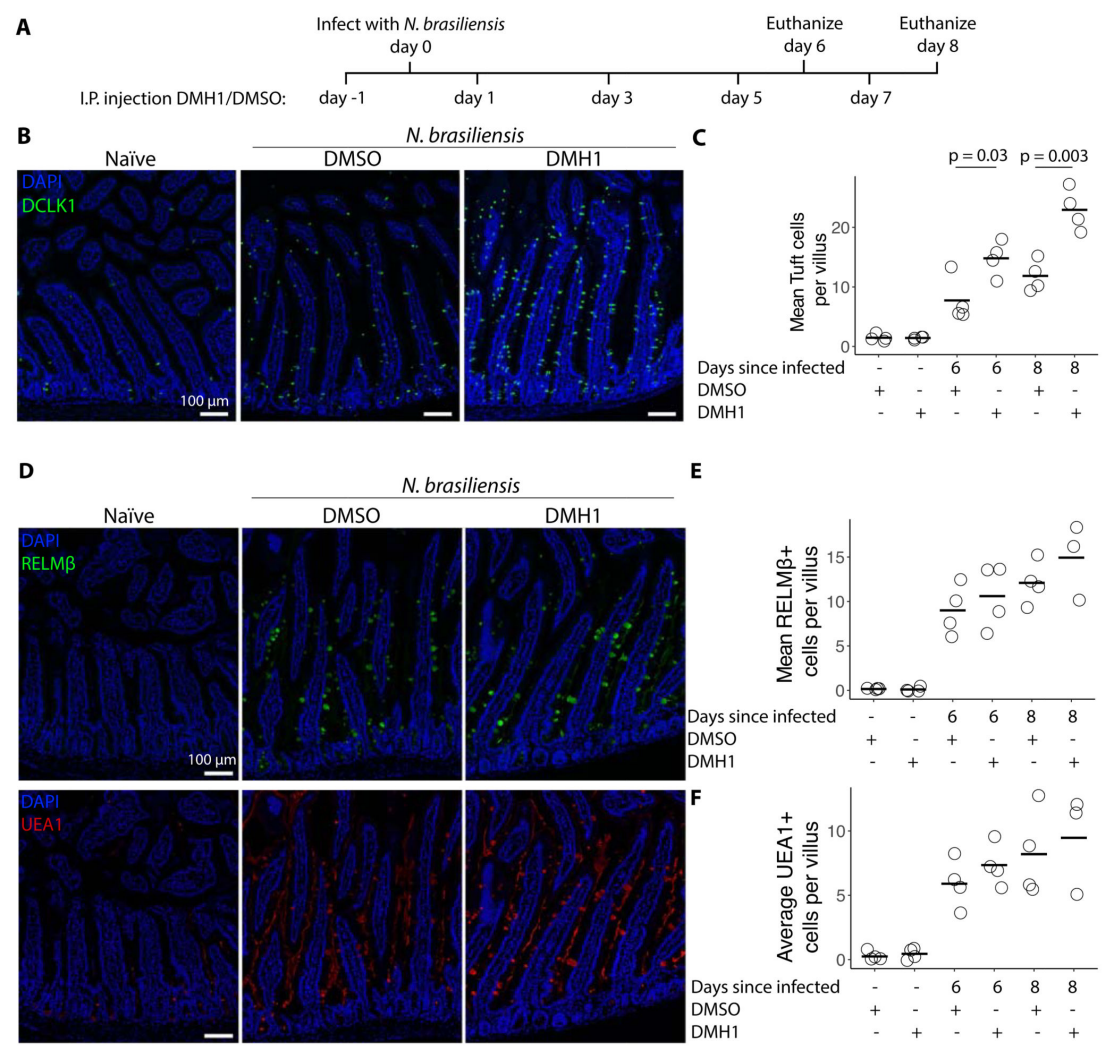
BMP signaling restricts *N. brasiliensis* induced tuft cell expansion *in vivo.* **A**, Timeline of *N. brasiliensis* infection and intraperitoneal injection of 5 μM DMH1 dissolved in DMSO or solvent alone. **B**, DCLK1 antibody staining of duodenum from mice 8 days after infection with *N. brasiliensis* and injected with DMH1 according to timeline shown in A. **C**, Quantification of images shown in B and additional images. Each dot represents one mouse. Statistics calculated with unpaired two tailed T-test. **D**, RELMβ antibody and UEA1 staining of duodenum from mice 8 days after infection with *N. brasiliensis* and injected with DMH1 according to timeline shown in A. **E, F**, Quantification of images shown in D and additonal images. Each dot represents one mouse.

Organoid work allows us to study intestinal epithelial mechanisms in isolation. However, *in vivo* there are many additional cell types that together orchestrate immunity to infection. To investigate the role of ALK2 signaling in *N. brasiliensis*-induced tuft cell hyperplasia, we injected mice with either DMH1 or its solvent (DMSO) intraperitoneally every other day starting one day before infection (Fig. 6A). Confocal staining of DCLK1 revealed an increase in the number of tuft cells at both day 6 and day 8 after infection when comparing DMH1-treated to DMSO-treated animals (Fig. 6B,C). The increase in tuft cells was also associated with a non-significant increase in *Il25* but not *Il13* (Fig. S9A). No difference was found in RELMβ or UEA1 (goblet) positive cells, indicating that the difference is specific to tuft cells (Fig. 6D-F).

**Figure 7:**
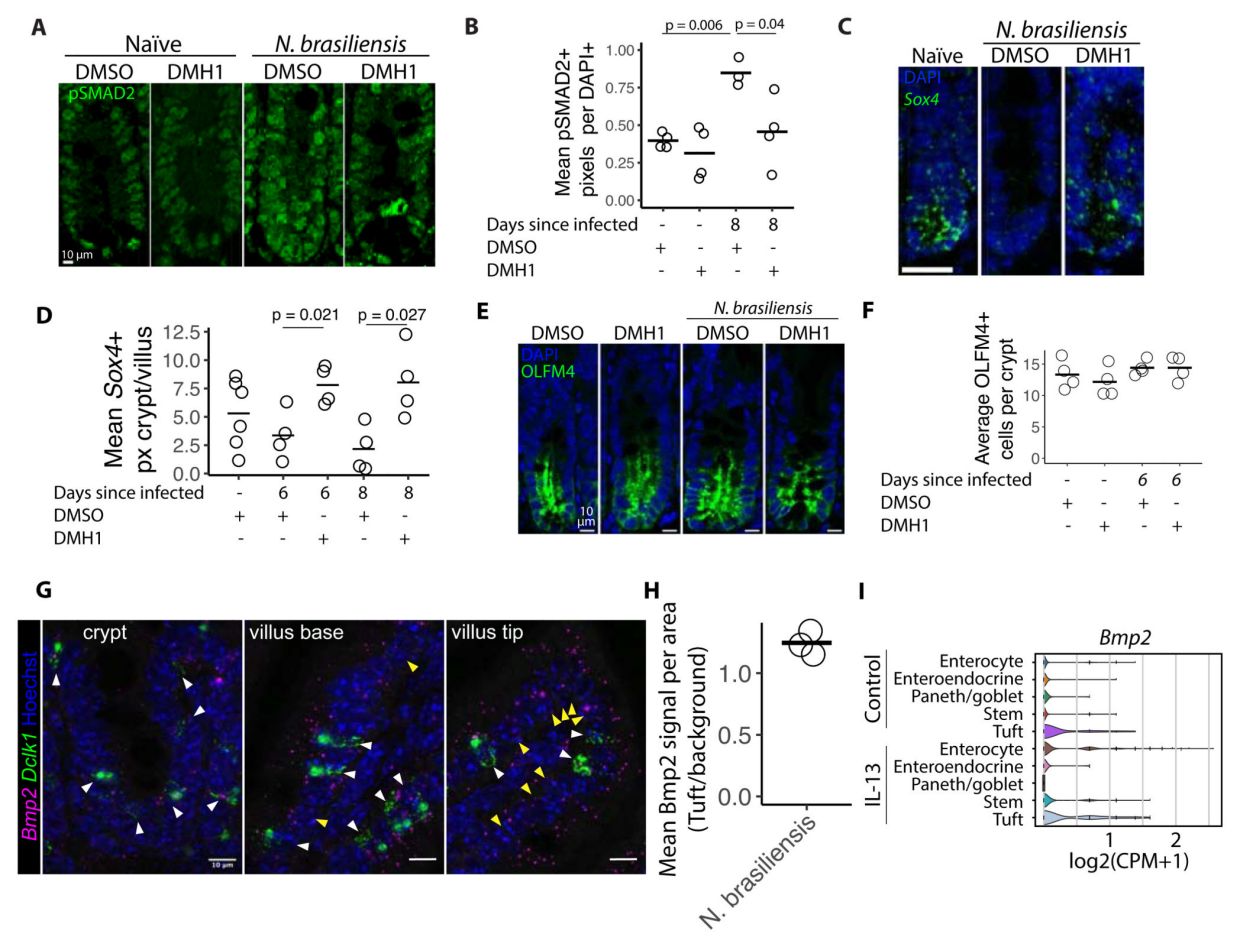
DMH1 restricts *N.brasiliensis* induced BMP signalling. **A**, pSMAD2 antibody staining in crypts from duodenum from mice 8 days after infection with *N. brasiliensis.* DMSO or DMH1 was injected every other day starting one day before infection. **B**, Quantification of images shown in A. Each circle represents the mean of at least 5 crypts in one mouse measuring pSMAD2+ pixels divided by DAPI+ pixels. Statistics calculated with unpaired two tailed T-test. **C**, RNAscope probe for *Sox4* in in crypts from duodenum 8 days after infection. Scale bar is 25 μm. **D**, Quantification of images shown in C and additional images. Each circle represents the mean of at least 5 crypts in one mouse measuring *Sox4*+ pixels in the crypt divided by *Sox4*+ pixels in the villus. Statistics calculated with unpaired two tailed T-test. **E**, Confocal images of OLFM4 staining in in crypts from duodenum 6 days after infection. **F**, Quantification of images shown in E. Each dot represents the average of at least 5 crypts from one mouse. **G**, RNAscope probe for *Bmp2* in indicated tissue from duodenum 6 days after infection. **H**, Quantification of crypts from images shown in G. *Bmp2* signal per area was measured in the immediate vicinity of *Dclk1* signal divided by cells not positive for *Dclk1* (defined as background). Both tuft cell area and background area was manually defined and each circle represents one mouse. **I**, Violin plots of gene expression from scRNAseq data of small intestinal organoids treated with IL-13 and untreated from Biton et al (3).

To test if BMP/TGF-β signalling is altered during infection we performed pSMAD2 staining (Fig. 7A,B). We found that there was a significant induction of nuclear pSMAD2 levels in crypt cells of infected mice compared to uninfected mice, and that this induction required ALK2 signalling as it did not occur in DMH1-treated mice (Fig. 7A,B). Based on our organoid work, we proposed that BMP/TGF-β signalling leads to repression of *Sox4* (Fig. 5G,I,J). Indeed we find a reduction of crypt-base located *Sox4* levels after infection and this repression did not occur in DMH1-treated animals (Fig. 7C,D). As reported by others, we found *Sox4* enriched at the stem cell zone (26, 27), however, the reduction of *Sox4* did not lead to a reduction of numbers of intestinal stem cells, as measured by counting OLFM4+ cells in crypts (Fig. 7E,F). Nor did it affect crypt proliferation as assessed by Ki67 staining (Fig. S9B,C) or apoptosis as assessed with cleaved Caspase3 staining (Fig. S9D,E). Finally, we investigated the association between *Bmp2* and tuft cells *in vivo* by using RNAscope probes for *Bmp2* and *Dclk1* we found *Bmp2* to be associated with tuft cells in the crypt (Fig. 7G,H). This association is not exclusive to tuft cells as we do see *Bmp2* in non-tuft cells, especially in the villus tip, a conclusion which is supported by the association of *Bmp2* with both enterocytes and tuft cells in organoids stimulated with IL-13 from scRNAseq data from (3) (Fig 7I). Taken together, these results suggest that BMP/TGF-β signaling restricts *N. brasiliensis* dependent tuft cell expansion *in vivo* by controlling *Sox4* expression.

## Discussion

The intestinal epithelium is capable of rapidly altering its cellular composition to defend against pathogens. Here, we provide a comprehensive comparison of how different immune responses mechanistically drive changes in the intestinal epithelium. Specifically, we find that key cytokines associated with different modes of immunity influence developmental pathways to guide changes in epithelial composition.

In recent years, tuft cells have been identified as regulators of intestinal health in general (9), and have taken center stage in the defense against helminth infections specifically (28). They express receptors that help them detect helminths, which together with type 2 cytokines results in their expansion (29, 30). This culminates in a feed forward loop where tuft cell derived IL-25 activates ILC2s and ILC2 derived IL-13 induces tuft cell differentiation (5–7). There are limits to this feed-forward loop though. For example, a putative epithelial-intrinsic PGD_2_-CRTH2 axis counteracts the IL-13-induced epithelial response (31), and ILC2-expressed CISH blocks ILC2 activation, which in turn limits tuft cell expansion (32). Other, competing, cytokines can also affect this loop as IL-22 inhibits IL-13 induced tuft cell responses (33). Finally, pathogens may also take part. Two studies recently showed that *Heligmosomoides polygyrus* secreted products can inhibit tuft cell expansion, likely by reprogramming epithelium towards a fetal-like state (34, 35).

Here, we identify that intestinal-epithelial intrinsic *Bmp* signaling can act as a brake on IL-13-induced tuft cell expansion (Fig. 4). We attribute this feedback mechanism to BMP/TGF-β signaling in general rather than a single BMP protein as we found both *Bmp2* and *Bmp8b* to be induced by IL-13 in organoids. In further support, the ALK5 inhibitor SB525334 show a similar ability as the ALK2 inhibitor DMH1 to increase tuft cell numbers upon IL-13 treatment. Our data indicate that the IL-13-mediated tuft cell hyperplasia is limited by BMP/TGF-β-dependent inhibition of *Sox4. Sox4* + tuft cell progenitors are thus initially expanded by IL-13, and then repressed by BMP signalling. It is paradoxical that inhibition of BMP signaling can affect intestinal epithelial stem cells since the crypt is surrounded by BMP antagonists (36). However, these BMP antagonists incompletely block BMP signaling as some pSMAD1/5/8 activation is still found in LGR5+ stem cells (20), and we observed a distinct increase of pSMAD2 in intestinal crypts upon infection with *N. brasiliensis* (Fig. 7). Furthermore, our experiments comparing IL-13 and IL-13+DMH1 are done in the presence of NOGGIN, a BMP inhibitor, indicating these crypt inhibitors only partly inhibit BMP signaling.

In addition to our findings regarding cytokine-driven tuft cell expansion, we also provide data suggesting that both IL-13 and IL-22 induce goblet cell gene signatures, but that these are different (Fig. 2). There have been conflicting reports in whether IL-22 influence goblet cell levels in intestinal epithelium. Some studies find that the number of MUC2+ cells are unchanged or reduced upon IL-22 treatment (37, 38), however, a study that over express the IL-22 inhibitor IL-22BP in the gut found a reduction in goblet cells (39) and IL-22 knock out mice infected with *N. brasiliensis* have reduction in goblet cell induction as measured by Periodic acid schiff (PAS) staining (40). Additionally, the goblet-cell effector RELMβ is critical for resistance against both bacterial and helminth infections (41–43). We hypothesize that the difference between IL-13 and IL-22 induced goblet cells is related to the type of immune response. Immunity to parasites requires a ‘weep and sweep’ response, in which goblet cells play an essential role by secreting mucus (weeping) to facilitate the expulsion of helminths (44), whereas other responses may rely less on the induction of mucus and expansion of goblet cell numbers. Instead, we find that IL-22 rapidly induces goblet-cell specific ER stress response genes, which corroborates recent work that identified a pathologically relevant role for IL-22 (45).

Overall, our approach of stimulating intestinal organoids with cytokines has revealed that developmental pathways underlie the cellular compositional changes that occur as part of immune responses taking place in the intestine. In support, a recent study showed that Il17ra signalling in intestinal stem cells induces ATOH1, thereby inducing secretory cells through NOTCH signalling (46). These changes have been confirmed using relevant *in vivo* models of infection or inflammation. It is important to balance both active immunity/inflammation with resolution of inflammation, and many immunopathologies reflect the importance of maintaining this balance. The discovery of an innate BMP-driven brake on IL-13 induced immune changes suggest that targeting this pathway may therefore be a useful tool to aid resolution of inflammation in clinically relevant diseases.

## Supplementary Material

Supplementary Material

## Data Availability

Code for brightfield and confocal analysis: https://github.com/havardtl/coco Sequencing data is deposited in the ArrayExpress database (http://www.ebi.ac.uk/arrayexpress) under the following accession numbers: RNAseq of small intestinal organoids treated with cytokines for 24 hours: E-MTAB-9182 RNAseq of duodenal intestinal epithelium from mice infected with *N. brasiliensis*: E-MTAB-9183 RNAseq of colon intestinal epithelium from mice infected with *C. rodentium*: E-MTAB-9184 RNAseq of small intestinal organoids treated for 72 hours with IL-13, IL-22 and DMH1: E-MTAB-9185 RNAseq of small intestinal organoids treated with IL-13 for 0h, 1h, 4h, 8h and 24h with and without DMH1: E-MTAB-10455 RNAseq of small intestinal organoids treated with IL-22 for 0h, 1h and 4h: E-MTAB-11273
