# Supplementary material for: BMP-signaling in the intestinal epithelium drives a critical feedback loop to restrain IL-13-driven tuft cell hyperplasia
Source: Sci Immunol. Author manuscript; Available in PMC 2023 Feb 1. (PMC7614132; doi:10.1126/sciimmunol.abl6543)
Supplement: Supplementary Material [file EMS163420-supplement-Supplementary_Material.pdf]

Supplementary file consisting of figures S1-S9 and methods

BMP-signaling in the intestinal epithelium drives a critical feedback loop to restrain IL-13-driven tuft cell hyperplasia

Håvard T. Lindholm[1,+*], Naveen Parmar[1,+], Claire Drurey[2], Marta Campillo Poveda[2], Pia Vornewald[1], Jenny Ostrop[1], Alberto Díez-Sanchez[1], Rick M. Maizels[2], and Menno J. Oudhoff[1,*]

[1]CEMIR – Centre of Molecular Inflammation Research, Department of Clinical and Molecular Medicine, NTNU – Norwegian University of Science and Technology, 7491 Trondheim, Norway

[2]Wellcome Centre for Integrative Parasitology, Institute of Infection, Immunology and Inflammation, University of Glasgow, G12 8TA Glasgow, United Kingdom.

[+]these authors contributed equally to this work

[*]corresponding authors: havard.t.lindholm@ntnu.no and menno.oudhoff@ntnu.no

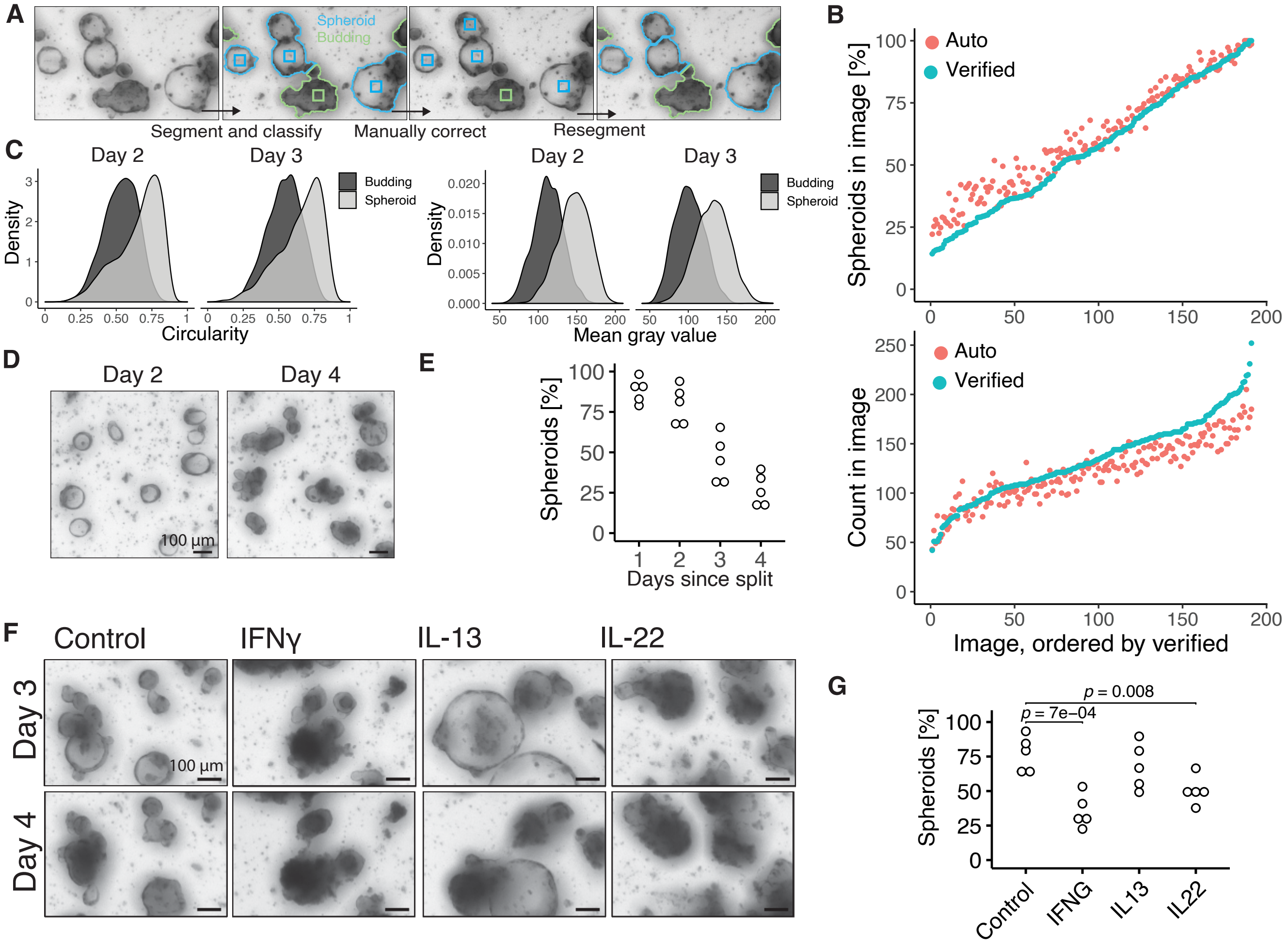

Figure S1: Image analysis pipeline. **A**, A segmentation algorithm is used to find the outline and center of an organoid and the segmented organoid is classified by a neural network. An optional manual correction of organoid centers and classification can be performed. The organoids can then be segmented again which gives an updated segmentation following the manual correction. **B**, Comparison of automatic segmentation and automatic classification of organoids compared to manually verified segmentation and manually verified classification. Each circle represents one image with approximately 100 organoids each. **C**, Distribution of circularity and mean gray value (higher value is whiter) of untreated small intestinal organoids. **D**, Representative images of untreated organoids. Media changed at day 2. **E**, Average fraction of spheroids in each image in same experiment as D. Each circle is one biological replicate and is the average of at least 3 images and in total this plot represents 16,462 organoids. **F**, Minimal projections of small intestinal organoids treated with cytokines. Days since seeding. **G**, Average fraction of spheroids at day 2 since splitting. Each circle is one biological replicate and is the average of at least 3 images and in total represents 10,462 organoids. P-values calculated with unpaired two-tailed T-test.

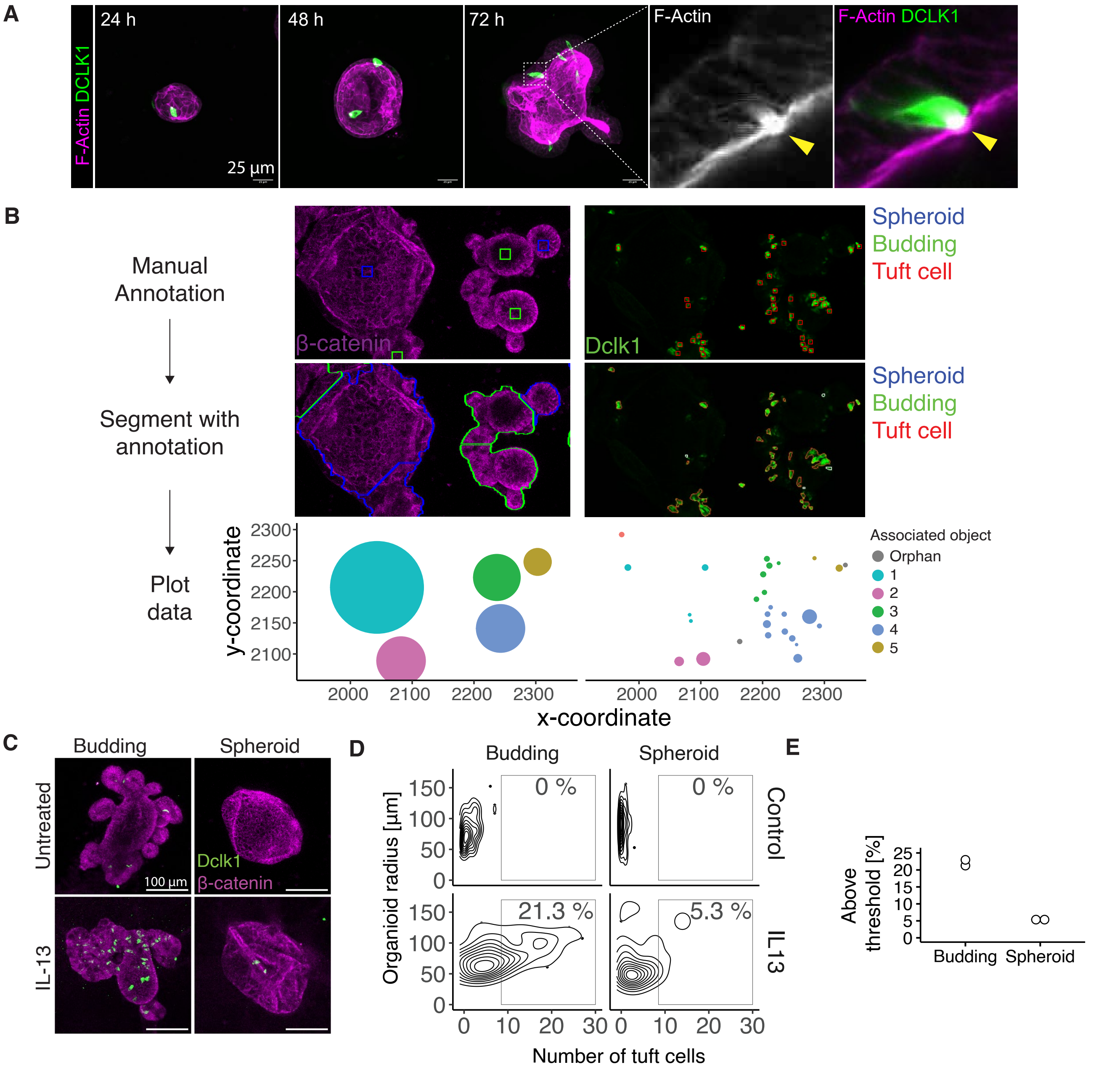

Figure S2: Quantification of number of tuft cells in organoids. **A**, Time course of tuft cell development in organoids. Times are hours since seeding. **B**, Image output from each step in the confocal image analysis pipeline. The center of each object of interest is marked with a square in a custom graphical user interface, and this information is used to separate overlapping objects. Furthermore, objects in other channels, like tuft cells, are analyzed to map which organoid they are inside. This information is summarized and used to plot number of tuft cells per organoid. **C**, Representative confocal images of small intestinal organoids at day 3 since splitting. **D**, Quantification of images shown in C. Percentages are relative to total number of organoids in that treatment. Plot shows the distribution of 192 organoids. **E**, Percentages for IL-13 treated organoids calculated as shown in D. Each circle is one biological replicate. All images in this figure are projections of Z-stacks.

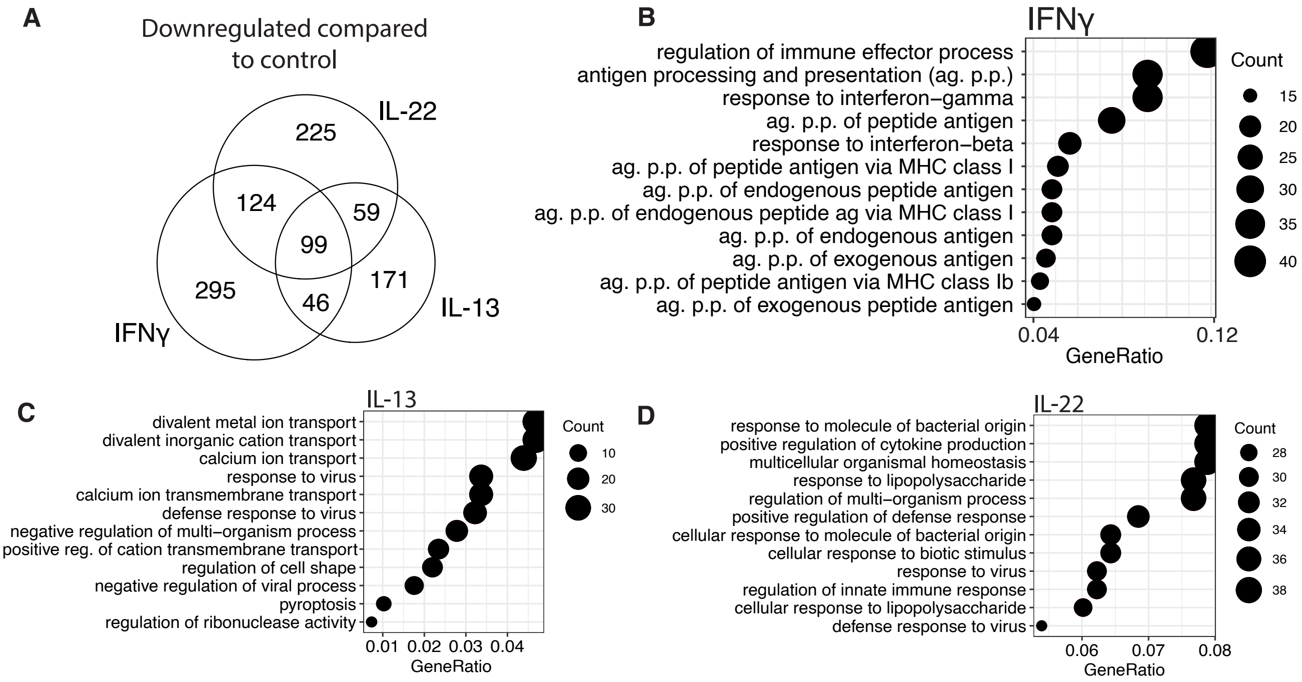

Figure S3: Details of RNA sequencing data of cytokines. **A**, Number of down-regulated significant genes from small intestinal organoids treated with indicated cytokine for 24 hours compared to control ($p<0.05$ and log2fc < -1). **B-D**, Top hits from GO-term analysis of "biological process" in RNAseq data from intestinal organoids treated with indicated cytokine for 24 hours compared to control.

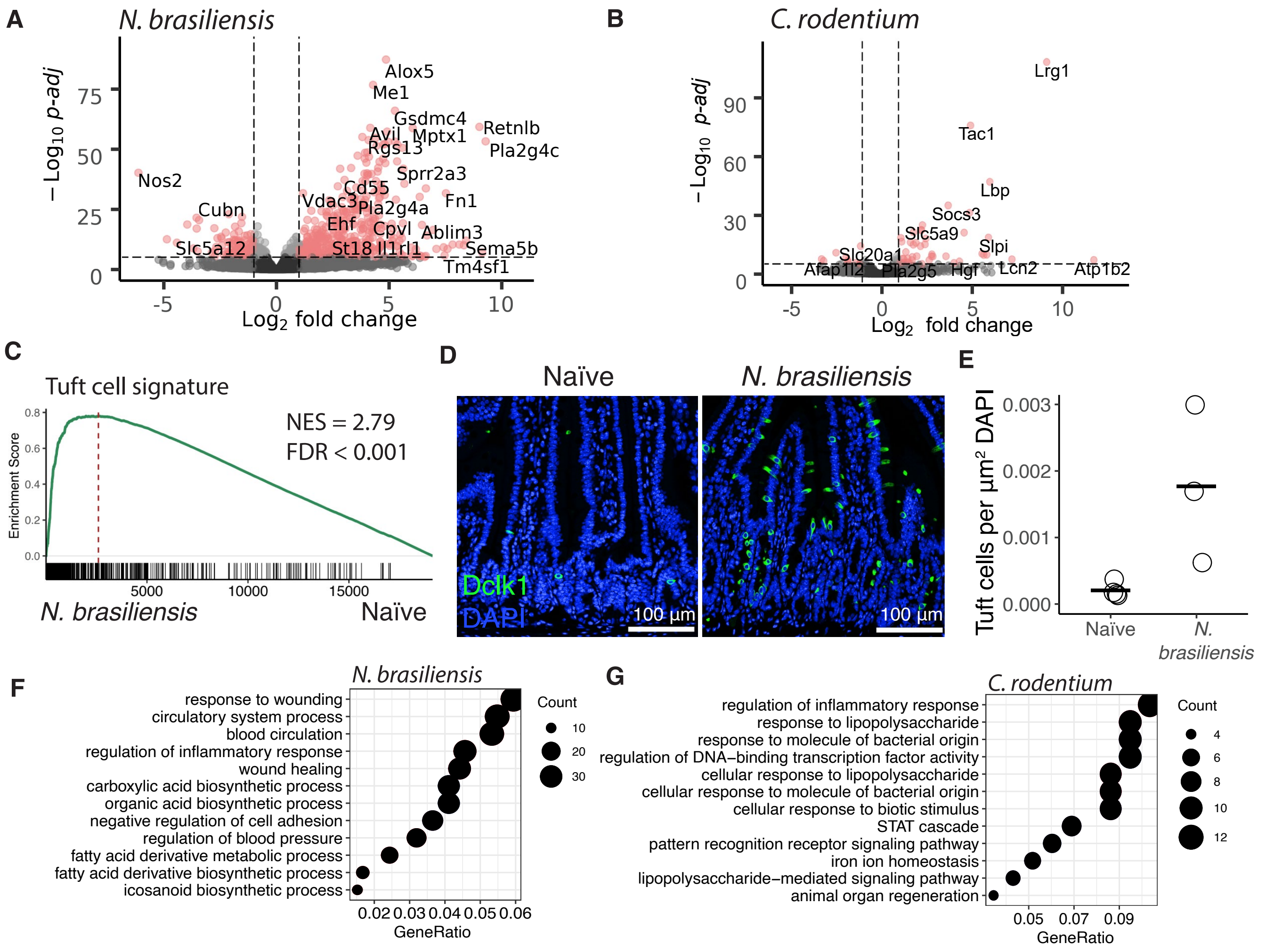

Figure S4: **A**, Volcano plot of RNAseq from intestinal epithelial tissue extracted from duodenum from mice seven days after infection with *N. brasiliensis.* **B**, Volcano plot of RNAseq from intestinal epithelial tissue extracted from colon from mice six days after infection with *C. rodentium.* **C**, GSEA analysis of a tuft cell signature on mRNA data from intestinal epithelium from *N. brasiliensis* infected mice. **D**, Confocal microscopy of cross section of mouse intestine seven days after infection with *N. brasiliensis* and naïve. **E**, Automatic tuft cell count from same experiment as D. Each circle represents quantitation from one full cross section from one mouse. **F**, **G**, Top hits from GO-term analysis of "biological process" in RNAseq data from mouse intestine infected with indicated pathogen.

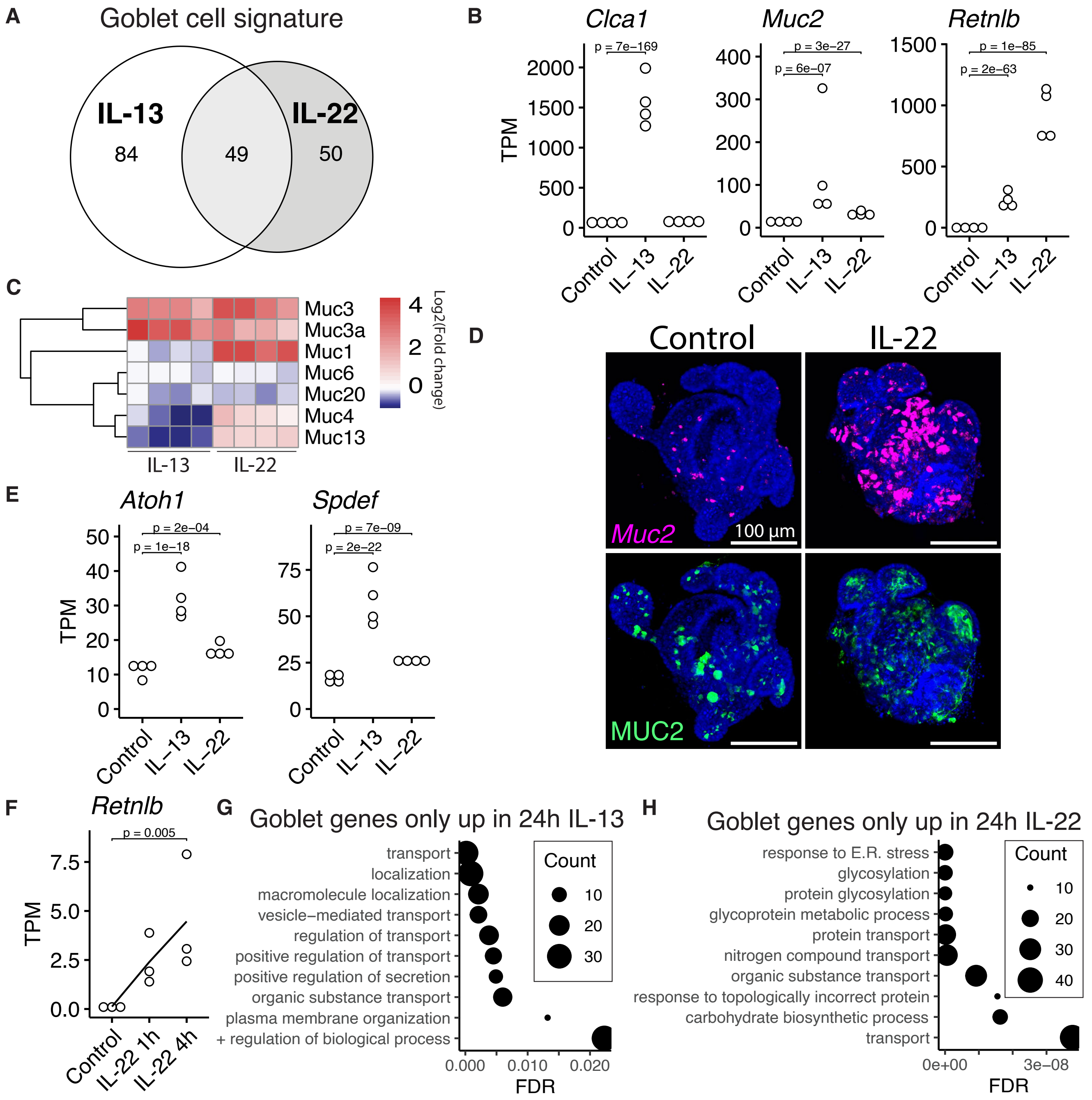

Figure S5: IL-13 and IL-22 gives different goblet cells also after 72 hours of cytokine stimulation. **A**, Distribution of how the plate based scRNAseq goblet cell gene set from Haber et al is changed upon 72 hours of IL-13 and IL-22 treatment. Up is defined as lg2fc>0.5 and p-adj<0.05. **B**, Gene expression from intestinal organoids treated for 72 hours with indicated cytokine. Each circle is one biological replicate. Statistics calculated with DESeq2, see methods. **C**, Expression of mucins in intestinal organoids stimulated IL-13 or IL-22 for 24 hours compared to control. **D**, Confocal imaging of organoids stained with an antibody staining for MUC2 in green and RNAscope probe for *Muc2* in purple. Organoids were imaged 3 days after seeding and 5 ng/mL of IL-22 was used since day of seeding. **E**, See B. **F**, Gene expression from intestinal organoids treated for 1 and 4 hours with IL-22. Each circle is one biological replicate. Statistics calculated with DESeq2, see methods. **G**,**H**, GO terms associated with gene sets seen in venndiagram in figure 2B. FDR = False discovery rate.

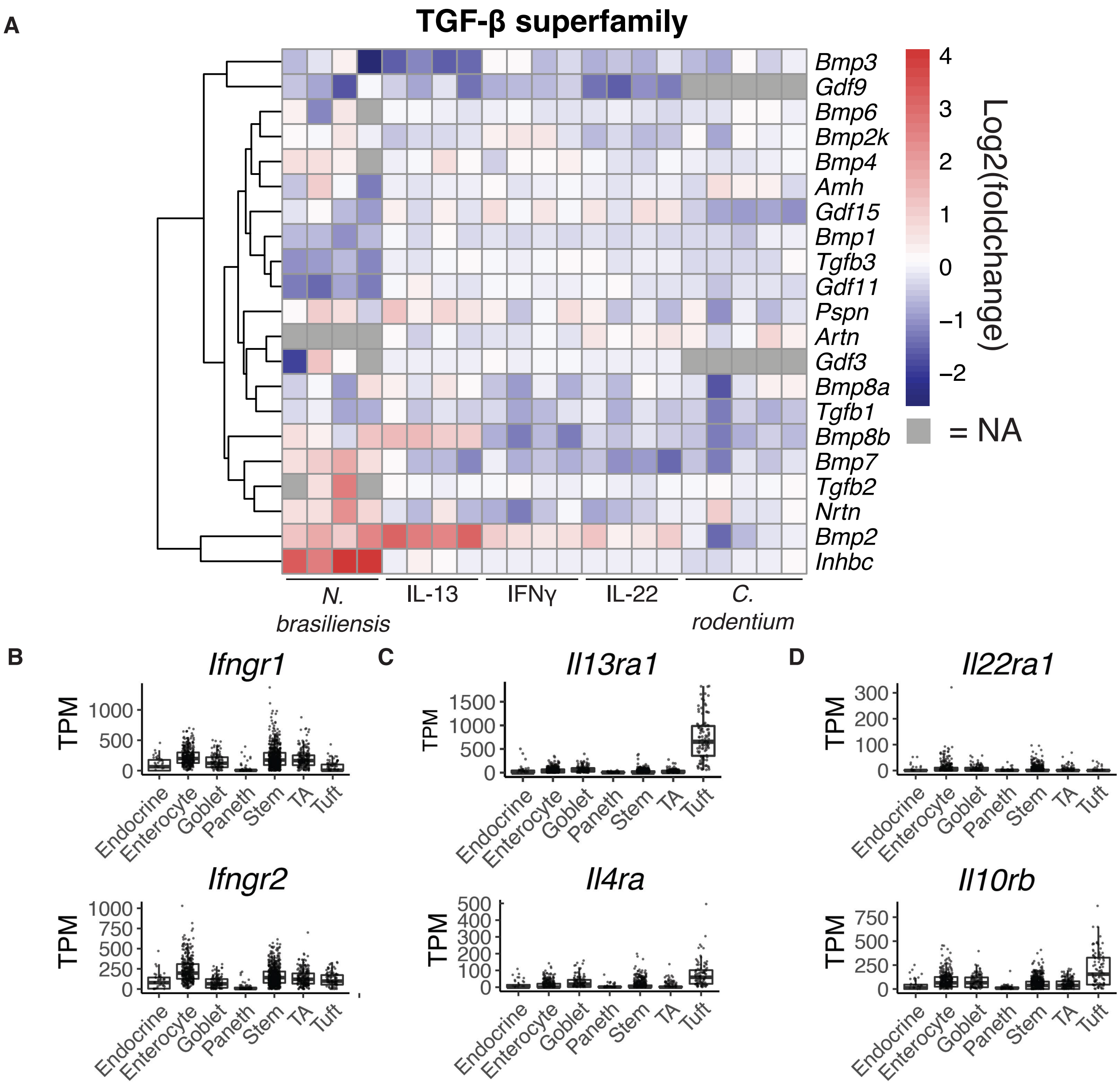

Figure S6: *Bmp2* is the main TGF-β superfamily member induced by IL-13. **A**, Heatmap of log2 fold change for TGF-β superfamily members from mRNAseq data from intestinal epithelium from mice infected with *N. brasiliensis* or *C. rodentium* and organoids treated for 24 hours with indicated cytokine. **B-D**, Expression of cytokine receptors, for IFNγ (B), IL-13 (C) and IL-22 (D), from plate based scRNAseq from Haber et al (*1*). TA = Transit amplifying.

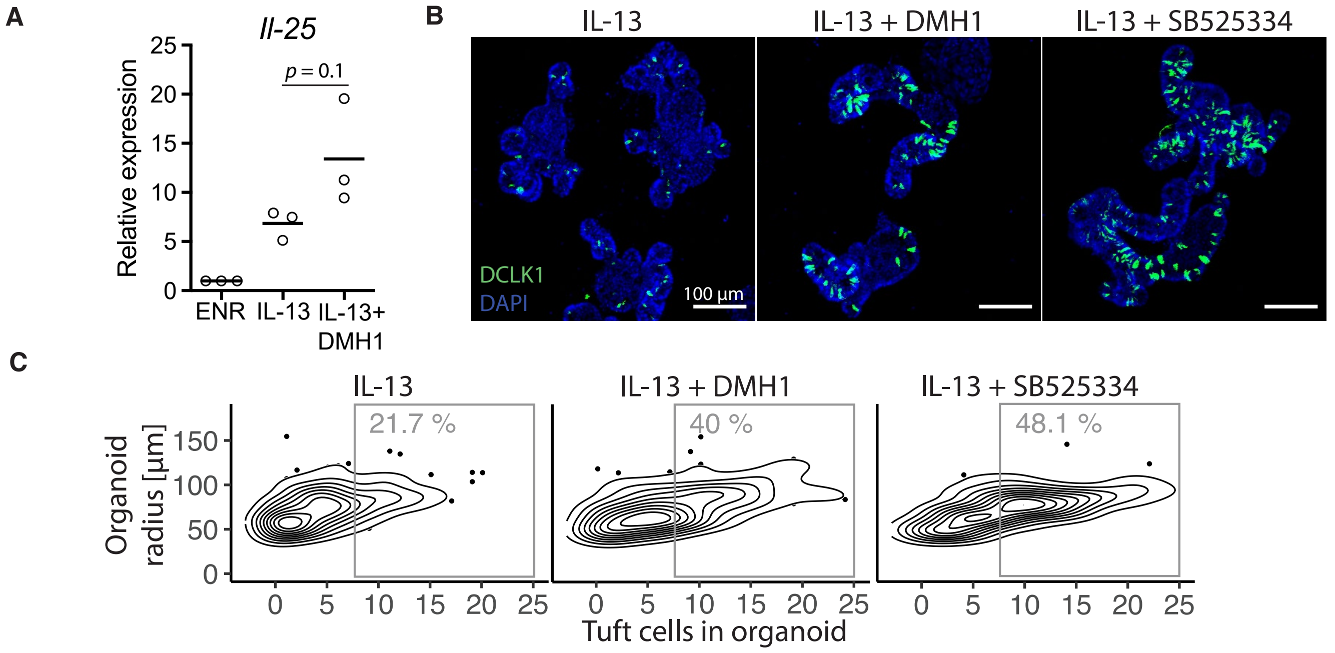

Figure S7: The effect of SB525334 on IL-13 dependent tuft cell hyperplasia. **A**, Relative expression of *Il-25* in intestinal organoids treated with indicated treatment for 72 hours determined with qPCR. p-value is calculated with unpaired two tailed T-test. **B**, Confocal staining of DCLK1 in small intestinal organoids treated with indicated treatments for 72 hours. **C**, Automatic quantification of same experiment as B. Each dot is one organoid.

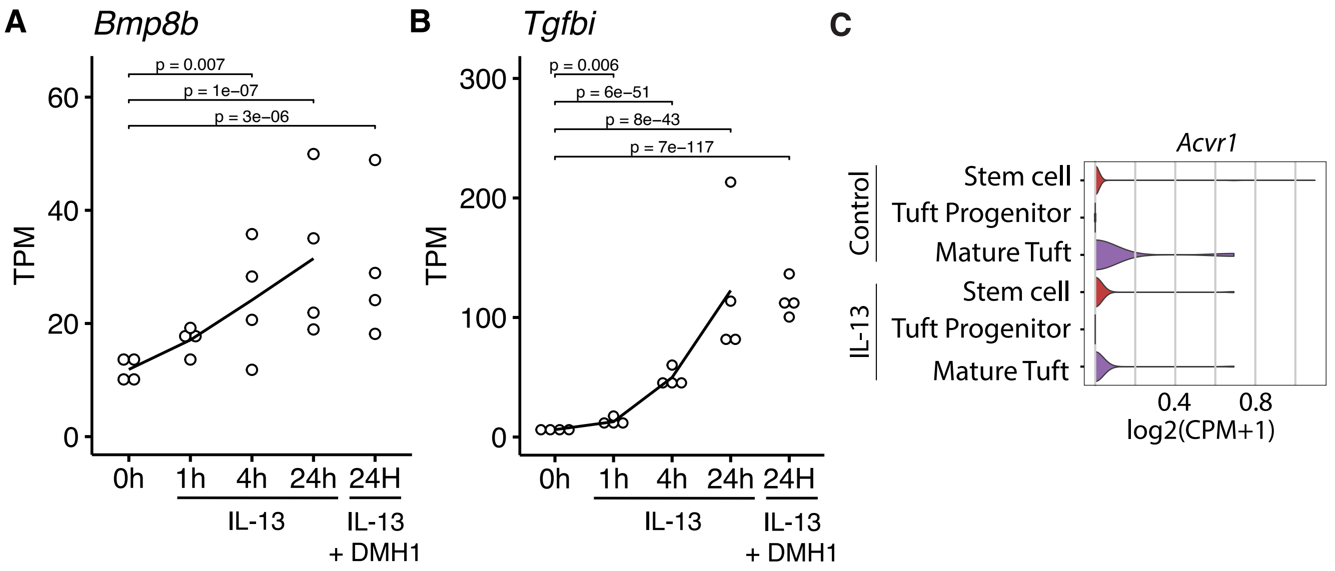

Figure S8: Time course of *BMP8b* and *Tgfbi* expression. **A**,**B**, Time points for RNAseq experiment from small intestinal organoids treated with 10 ng/mL IL-13 at indicated timepoints and with and without 5 µM DMH1 for 24 hours. Each circle is one biological replicate, statistics calculated with DEseq2, see methods. **C**, Violin plots of gene expression from scRNAseq data of small intestinal organoids treated with IL-13 and untreated from Biton et al (*2*).

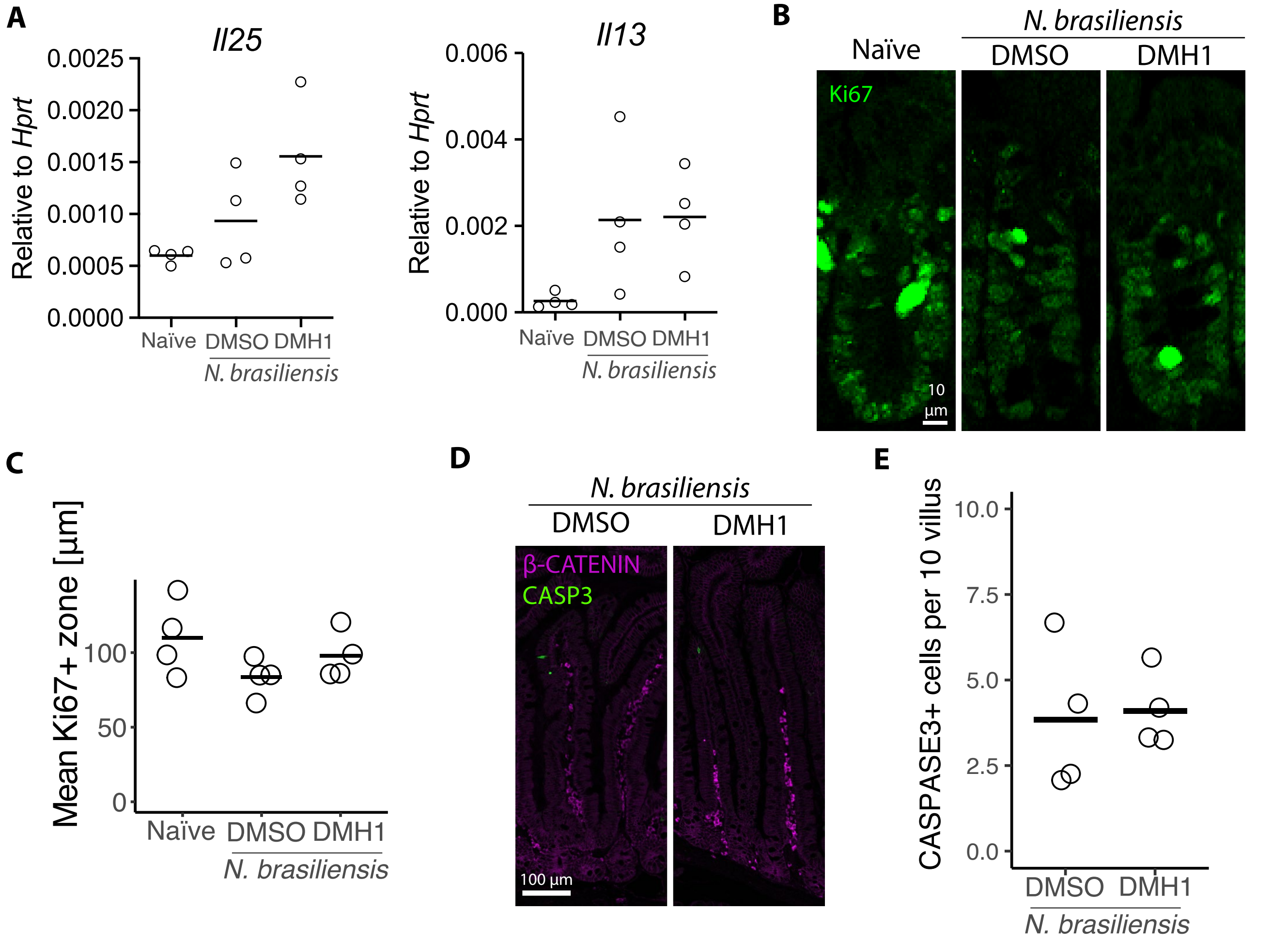

Figure S9: Further characterization of mice treated with DMH1 and DMSO and infected with *N. brasiliensis*. **A**, qPCR of indicated gene in duodenal intestinal tissue after 8 days of infection with *N. brasiliensis*. **B**, Ki67 staining in duodenal crypts from mice after 8 days of infection with *N. brasiliensis*. **C**, Quantification of height of Ki67+ zone in images shown in B. Each dot is the average of at least 5 crypts in one mouse. **D**, Staining of cleaved Caspase3 in duodenal crypt and villus from mice after 8 days of infection with *N. brasiliensis*. **E**, Manual quantification of images shown in D. Each circle is the average of at least 20 villus in one mouse.

Methods

Animal experiments

All mouse work done in Norway was performed in accordance with CoMed NTNU institutional guidelines and Norwegian legislation on animal experiments. Mice were handled under pathogen-free conditions. The C57BL/6 mice strain was used for all experiments.

The mice studies for *Citrobacter rodentium* infection were approved by Norwegian Food Safety Authority (FOTS ID: 11842). Briefly, C. rodentium was grown at 37 °C in Luria- Bertani (LB) medium supplemented with chloramphenicol (30 µg/ml) overnight. 9-11 weeks old mice were orally gavaged with a culture of $10^8 - 10^9$ CFU per mouse delivered in a volume of 100 µL of sterile PBS. Mice remained infected with *C. rodentium* for six days. To determine the infection level, fecal samples were collected on day 5, homogenized in sterile PBS with a FastPrep homogenizer. After serial dilution of fecal samples, homogenates were plated on chloramphenicol resistant agar plates and the plates were counted after 24 h of growth at 37 °C. After 6 days of infection, mice were euthanized by CO_2 inhalation, parts of distal colon tissue was harvested for isolation of crypts as described below. RNA was isolated and samples were further used for RNAseq analysis.

Mice studies with *Nippostrongylus brasiliensis* infection used mice aged 8-12 weeks maintained in individually ventilated cages according to UK Home Office guidelines. *N. brasiliensis* was maintained as previously described (*3*). Mice were infected with larva of *N. brasiliensis* subcutaneously. 200 L3 larvae was used for sequencing experiments and 400 L3 larvae was used for experiments with DMH1. For experiments with DMH1, 4 mg/kg DMH1 dissolved in 20 µL DMSO, or control of DMSO alone, was administrated every other day, starting one day before infection. Mice were euthanized via cervical dislocation, and duodenal tissue harvested to use in imaging and for isolation of crypts as described below.

Small intestine and colon crypt isolation

Crypts from small intestine and colon where isolated according to a published protocol (*4*). In summary, the duodenal small intestine (first 10 cm from end of stomach) or distal colon of mouse was washed in ice-cold PBS and opened longitudinally. A microscopy cover slip was used to gently scrape of excess mucus and villus before the intestine was cut into 2-4 mm pieces. The fragments were resuspended in 30 mL ice-cold PBS and pipetted up and down with a 10 mL pipette. Supernatant was discarded and this step was repeated 5-10 times until the supernatant was clear. The fragments were incubated in 2 mM EDTA in PBS for small intestine, and 10 mM EDTA in PBS for colon, at 4 °C for 30 min with gentle rocking. The supernatant was removed, 20 mL of ice-cold PBS was added and the fragments were pipetted up and down. This washing step was repeated until the crypt fraction appeared as seen in a microscope. The 1-3 consecutive crypt fractions were passed through a 70 µm cell strainer and collected into a FCS coated 50 mL tube. The crypts were spun down at 300 x*g* for 5 min and washed once in 10 mL ice cold PBS at 200 x*g* for 5 min.

Small intestinal organoid culture

Approximately 200-500 crypts were resuspended in 50 µL Matrigel (Corning, 734-1101) and kept at 4 °C. 50 µL of the Matrigel solution was added to the center of a pre-warmed 24 well plate or 8-well microscopy slide (Ibidi, 80821) and quickly transferred to an incubator at 37 degrees with 5 % CO_2. After 5 min the pellet had solidified and 500 µL of basal culture medium was added and the plate was put back into the incubator. Basal culture medium consisted of advanced Dulbecco's modified Eagle medium - F12 supplemented with penicillin/streptomycin, 10 mM HEPES, 2 mM Glutamax, 1x N2 [ThermoFisher Scientific 100X, 17502048], 1x B-27 [ThermoFisher Scientific 50X, 17504044], and 1x N-acetyl-L-cysteine [Sigma, A7250]) and overlaid with ENR factors containing 50 ng/ml of murine EGF [Thermo Fisher Scientific, PMG8041], 20 % condition medium (CM) from a cell line producing R-Spondin (kind gift from Calvin Kuo, Stanford University School of Medicine, Stanford, CA, USA) and 10 % Noggin-CM (a kind gift from Hans Clevers, Hubrecht

Institute, Utrecht, The Netherlands). The culture medium was replaced every 2-3 days. Organoids were passaged by disrupting them with strong mechanical pipetting, letting the solution cool on ice before centrifuging at 200 x*g*, 5 min at 4 °C and resuspending in Matrigel. Organoid lines were maintained and normal budding morphology was observed throughout passaging. All experiments have been done within the range of 4-50 passaging events.

ELISA

BMP2 ELISA

qPCR

qPCR of intestinal organoids treated with IL-13 + DMH1 and nippo RNAlater tissue

Bright field imaging and quantification of intestinal organoids

Z-stacks covering the entire Matrigel droplet were captured using a EVOS2 microscope with CO_2, temperature and a humidity-controlled incubation chamber (Thermo-Fisher Scientific). 2D morphological properties of organoid objects as well as their classification were gathered using a custom analysis program written in python based on opencv2 (*5*). The brightness of images were autoscaled to max brightness, and a canny edge detection algorithm was run on each individual z-plane using the opencv2.canny function. Groups of pixels below a certain size was removed and a minimal projection of the edges was generated. This image was used to define the contour of objects. The implementation of opencv2s wathershed algorithm was used to split somewhat overlapping objects from each other and the center of the object was defined as the pixel furthest from the edge of the object. Each defined object contour was used to extract a 120x120 pixel picture of the object on a white background from a minimal projection of the original z-stack. These images were used to classify the organoid as either "Junk", "Budding" or "Spheroid" with a convolutional neural network implemented using Tensorflow and Keras (*6*, *7*). The network

was trained on about 25 000 manually classified 120x120 images prepared as just described. For initial analysis these data were plotted, but for plots used in this publication the pictures were reviewed manually in a graphical user interface (GUI) written in python. This GUI displayed the center of each object and its classification and enabled addition of new object centers or editing of automatically found object centers. The manually verified annotations were used to re-run the segmentation as described above, but this time the classification of the objects were not changed and a watershed algorithm were used to segment object contours based on the manually curated object centers. Finally, objects with a size less than 300 pixels were filtered out. To highlight differences between treatments, thresholds for percentage calculations were generally chosen to be set around the upper bound of the treatment with the lowest organoid radius. Plots were made with the R-package ggplot2 (*8*).

Immunofluorescence staining of organoids and imaging

The Matrigel used for organoids grown for immunofluorescent imaging were cultured in Matrigel mixed with 25 % basal culture medium (described above) in eight-well slides (Ibidi, 80821). Media was removed and organoids were fixed in 300 µL 4% PFA with 2 % sucrose for 30 minutes. Fixation solution was removed and 300 µL PBS was added, after 5 minutes the PBS was removed. This washing step was done a total of two times. The PBS was removed and 0.2% Triton X-100 in PBS was added for 30 minutes at room temperature to permeabilize the cell membranes. The wells were then washed in PBS, 3 times, 5 minutes each time. Free aldehydes were blocked in 100 mM Glycine for 1 hour at room temperature and thereafter the wells were incubated in 300 µL blocking buffer (2 % normal goat serum, 1 % BSA and 0.2 % Triton X-100 in PBS) for 1 hour at room temperature. Primary antibodies, β-catenin (1:200, mouse mAb, Santa Cruz Biotechnology, sc-7963), DCLK1 (1:250, rabbit pAb, abcam, ab31704), MUC2 (1:200, rabbit pAb, Santa Cruz Biotechnology sc-15334) and RELM-β (1:200, rabbit pAb, PeproTech 500-P215) were diluted in 150 µL blocking buffer and incubated overnight at 4 °C. The next day samples were washed in PBS with agitation, 3 times 10 minutes each time. The appropiate Alexa fluor Secondary antibody

(1:500), and DAPI (1:10 000) and UEA1 (Ulex Europaeus Agglutinin I Rhodamine-labeled Dil 1:500, Vector laboratories RL1062) was added in blocking buffer (1 % normal goat serum, 0.5 % BSA and 0.2 % Triton X-100 in PBS) and incubated at 4 °C with agitation over night. The next day the samples were washed in PBS, 3 times 5 minutes each time, and 250 µL Fluoromount G medium (ThermoFisher Scientific, 00-4958-02) was added to the well. Z-stacks of tile-scans covering the entire well were acquired on a Zeiss Airyscan confocal microscope using a 10X objective lens.

Immunofluorescence staining of tissue sections and imaging

The harvested intestinal tissue was fixed with 10% formalin for 48 h at room temperature. Tissue was subsequently dehydrated through a series of graded ethanol and then embedded in paraffin wax blocks. 5 µm thick sections were cut using a microtome, floated in a 40 °C water bath and transferred to a glass slide. After drying, the tissue sections were kept in an oven at 60 °C for 30 minutes and deparaffinized in two changes of Neo-clear (Xylene substitute) for 5 min each, followed by graded ethanol (100 % ethanol 2 times with 3 min each, and next transfer once through 95 %, 80 %, and 70 % ethanol and water 3 min each). Heat mediated antigen retrieval was performed using citrate buffer except for pSmad2 (phospho S255) where Tris/EDTA buffer pH 9 was used. Tissue sections on glass slides were marked with hydrophobic pen (PAP pen, ab2601) to keep staining reagents on the tissue section. Next, blocking buffer (1% BSA, 2% normal goat serum, 0.2% Triton X-100 in PBS) was added onto the sections of the slides and incubated in a humidified chamber at room temperature for 1 hour. Appropriately diluted primary antibody (antibody dilution buffer, e.g. 0.5 % bovine serum albumin, 1 % normal goat serum, 0.05 % Tween 20 in PBS) was added to the sections on the slides and incubate in a humidified chamber at 4 °C overnight. Primary antibodies used exclusively for tissue where Ki67 (1:100, rabbit mAb, invitrogen MA5-14520), pSMAD2 (phospho S255) (1:100, rabbit mAb, abcam ab188334), OLFM4 (1:100, rabbit mAb, Cell signaling 39141S). See organoid staining section for details of the other antibodies. The slides were washed in 0.2 % Triton X-100 10 min each. Next, secondary antibody conjugated with fluorochromes was added to the slides for 1 hour at room temperature in a humidified chamber.

DAPI and UEA1 was added to stain goblet cells. After rinsing three times with PBST (0.2 % Triton X-100 in PBS) for 10 min each, slides were mounted in Fluoromount G. Complete tile scans were acquired with a Zeiss Airyscan confocal microscope, using a 10x or 20x objective lens.

Combined RNA Scope and Immunofluorescence in organoids

Organoids were cultured in 8 Well Chamber, removable (IBIDI, Catalog no.80841) and ACD RNAScope protocol was followed with a few modifications. After treatments, media was removed, and 10 % neutral buffered formalin (NBF) was added to wells for 30 mins at room temperature (RT). NBF was decanted and organoids were washed with PBS two times. For dehydration, 1X PBS was removed and 50 % ethanol was added for 1 minute, followed by 70 % ethanol and then 100 % ethanol. For re-hydration, 70 % ethanol was added for 1 minute followed by 50 % ethanol for 1 minute at RT. Before beginning the RNA scope steps, a hybridization oven was set to 40° C. Chambers were carefully removed from the slides and organoid area was marked with a RNA scope hydrophobic pen. Next, RNAscope hydrogen peroxide solution was added to the wells for 10 minutes at RT and then removed. Wells were washed with fresh distilled water for two times. Then, two drops of protease III (1:15) was added to each well for 10 minutes at RT. Protease III solution was removed, and wells were washed twice with PBS for 2 minutes. Probes were warmed in incubator at 40° C for 10 minutes. Next, *Muc2* (ACD 315451-C3) probe (1: 100) was diluted in probe diluent (ACD 300041) and added to the wells for 2 hours in a hybridization oven kept at 40° C. After 2 hours, liquid was removed, and 1X wash buffer was added twice for 2 minutes. Next, 2 drops of RNAscope multiplex FL v2 Amp1 was added to each well for 30 minutes at 40° C in a hybridization oven. Wells were washed twice with 1X wash buffer. Next, RNAscope multiplex FL v2 Amp2 was added to the wells and incubated for 30 minutes at 40° C. Wells were washed twice with 1X wash buffer. After this, RNAscope multiplex FL v2 Amp3 was added to wells and incubated for 15 minutes at 40° C in a hybridization oven. After incubation, wells were washed twice with 1X wash buffer for 2 minutes. For *Muc2* probe, only HRP- C3 signal was developed. Next, 2 drops of RNAscope Multiplex FL v2 HRP-C3 was added to wells and incubated for 15

minutes at 40° C in a hybridization oven. Wells were washed twice with 1 x wash buffer for 2 minutes at RT. Next, Opal 690 (1:1000) diluted in TSA buffer was added to wells for 30 minutes at 40° C in a hybridization oven. Wells were washed twice with 1X wash buffer for 2 minutes at RT. Next, RNAscope Multiplex FL v2 HRP blocker was added to wells for 15 minutes at 40° C in a hybridization oven. Wells were washed twice with 1x wash buffer at RT. Next, 2 % Normal goat serum and 1 % BSA in PBS was added to wells for 30 minutes at RT. After this, RELMβ primary antibody (1: 200) and MUC2 primary antibody (1: 200) was added to wells overnight at 4° C in dark humidified chamber. Next day, wells were twice washed with PBS and anti-rabbit Alexa fluor 488 (1: 500), DAPI (1:1000) and UEA1-Rhodamine (1: 500) was added to wells overnight at 4° C in dark humidified chamber. Next day, solution was removed and washed twice with PBS. Slides were mounted with Fluoromount G and covered with cover slips. Images were acquired using Zeiss LSM 880 with a 20X objective.

RNA scope in tissue sections

RNA scope in tissue slides was performed according to RNAscope Multiplex Fluorescent V2 Assay manufactured by Advanced Cell Diagnostics. Briefly, the tissue slides were baked in a dry oven for one hour at 60° C. Next, paraffin was removed by incubating slides in Neoclear (xylene substitute) for 5 minutes twice at RT. After this, slides were incubated twice in 100 % ethanol for 2 minutes at RT. Then, hydrogen peroxide was added to the slides for 10 minutes at RT. Next, antigen retrieval was performed using 400 mL of 1X Target Retrieval reagents. Slides were kept in a beaker with boiling 1X Target Retrieval reagents on a hot plate heater for 15 minutes. Next, slides were transferred to a rinse container with distilled water and rinsed for 15 seconds. After that, slides were transferred to 100 % ethanol for 3 minutes. After drying, a barrier was created around the tissue using a hydrophobic barrier pen. Slides were stored at RT overnight. The next day, the hybridization oven was set at 40° C. RNAscope protease plus drops were added to cover the whole tissue, and slides were incubated at 40° C for 30 minutes. Slides were washed twice with distilled water. Sox4-C2 (471381-C2) probe was warmed at 40° C for 10 minutes in an incubator and then

cooled to RT. Next, 50 µL of *Sox4* probe (1:50) diluted in probe diluent was hybridized onto each tissue section by incubating slides at 40° C for 2 hours. Also, a few drops of negative control probe were added to the two separate slides to check the specificity. After 2 hours, slides were washed twice with 1X wash buffer for 2 minutes at RT. Next, a few drops of RNAscope Multiplex FLv2 Amp1 were added to each slide and incubated for 30 minutes at 40° C. Slides were removed and washed twice with 1X wash buffer for 2 minutes at RT. Next, slides were incubated with RNAscope Multiplex FLv2 Amp2 for 30 minutes at 40 ° C, and slides were washed with 1X wash buffer for 2 minutes at RT. Then, slides were incubated with a few drops of RNAscope Multiplex FLv2 Amp3 for 15 minutes at 40°C. Slides were washed twice with 1X wash buffer for 2 minutes at RT. Next, excess liquid was removed, and slides were incubated with RNAscope Multiplex FLv2 HRP-C2 in the oven for 15 minutes at 40° C. Slides were removed and washed twice with 1X wash buffer for 2 minutes at RT. Next, Opal-690 diluted (1:1000) in TSA buffer was added onto the slides in the oven for 30 minutes at 40°C and then washed twice with 1X wash buffer for 2 minutes at RT. Slides were further incubated with RNAscope Multiplex FLv2 HRP- blocker for 15 minutes at 40° C and then washed with 1X wash buffer at RT for 2 minutes. Slides were counter-stained with DAPI (1:1000) and mounted in Fluoromount G and covered with glass slides. 20X tile scan images were acquired using the ZEISS LSM880 microscope.

Quantification of immunofluorescence images

2D morphological properties of intestinal organoid objects in confocal images were gathered using a custom analysis program written in python based on opencv2 (*5*). Maximal projections of each channel were created as well as a rgb composite of all channels, hereafter referred to as the combined channel. The center position of all intestinal organoids as well as their class as either "budding" or "spheroid" were manually annotated in the combined channel using a custom graphical user interface. A binary threshold individual to each channel was then applied to create channel masks and additionally, these masks were added together to a combined mask. Contours of objects were defined based on the outline in combined mask and they were split using a watershed algorithm

where the manual annotations were used as input. Only objects in the combined channel with a manual annotation were kept for further analysis. 2D morphological properties such as area, perimeter and circularity of each object in the combined channel was acquired as well as the mean intensity of each channel. Furthermore, tuft cells were manually annotated in the DCLK1 channel using the custom graphical user interface. The number of tuft cells inside each object in the combined channel could therefore be counted. To highlight differences between treatments, thresholds for percentage calculations were generally chosen to be set around the upper bound of the treatment with the lowest induction in tuft cells. These data were processed in R and plotted with the ggplot2 package (*8*).

The same analysis were applied to confocal images of tissue staining with some changes. A binary mask defining the tissue area to quantify, for example crypts, villus or both, was manually made for each tissue section. Furthermore, manual annotation of tuft cells was done for a selection of images using a custom written graphical user interface. The number of manual tuft cells inside the manually made tissue section mask was counted. In addition, both the mean intensity of each channel and the number of pixels above a channel-individual threshold was measured for each channel inside each marked region in the tissue mask. This enabled a calculation of number of manually counted tuft cells per μm^2 DAPI positive pixel and quantification of fluorescence intensity per crypt. Furthermore, a binary threshold of the DCLK1 channel allowed definition of automatically defined tuft cell objects. Whether a DCLK1 object was a tuft cell or noise could be confirmed by checking for presence of a manual tuft cell annotation. In this way, gates for selecting tuft cells where defined and used for images that were not manually annotated. These data were processed in R and plotted with the ggplot2 package (*8*).

A similar approach was used to quantify *Bmp2* RNAscope signal in tuft cells compared to background. A mask was manually drawn around each *Dclk1* positive area approximately encompassing a single tuft cell. Another mask (called background) was drawn around the rest of the cells in the crypt without *Dclk1* signal. These masks were used in the same script as described in the previous paragraph, further processed in R, and plotted with the ggplot2 package (*8*)

RNA isolation and sequencing

RNA was extracted with Quick-RNA Microprep Kit according to the manufacturers instructions (Zymo, R1050). Library preparation and sequencing for 24 hour cytokine samples was performed by NTNU Genomic Core facility. Library preparation was done with Lexogen SENSE mRNA kit and the library was sequenced on two Illumina NS500 HO flowcells, 75 bP single stranded. All other library preparation and sequencing was performed by Novogene (UK) Co. using the NEB Next® Ultra™ RNA Library Prep Kit. Samples were sequenced at 150 bp paired end using a Novaseq 6000 (Illumina).

Batch RNA sequencing analysis

Reads were aligned to the *Mus Musculus* genome build mm10 using the STAR aligner (*9*). The count of reads that aligned to each exon region of a gene in GENCODE annotation M18 of the mouse genome (*10*) was counted using featureCounts (*11*). Genes with a total count less than 10 across all samples were filtered out. A differential expression analysis was done with the R-package DESeq2 (*12*), and volcano plots were plotted with the R-package EnhancedVolcano (*13*). PCA analysis was performed with the scikit-learn package with the function sklearn.decomposition.PCA (*14*). GSEA analysis was run with the log2(fold change) calculated by DESeq2 as weights, 10000 permutations and otherwise default settings using the R-package clusterProfiler (*15*). GO term analysis was run using the function enrichGO in clusterProfiler. The R-packages pheatmap and eulerr where used to make heatmaps and venndiagrams, respectively (*16*, *17*). RNAseq of organoids grown in the absence and presence of Noggin can be found from the ArrayExpress accession E-MTAB-9181 and analysed as described in (*18*).

Single cell RNA sequencing analysis

Barcodes, genes and matrix files for control and IL-13 treated samples were downloaded from GEO accession GSE106510 (*2*). Additionally, BAM files from GSE106510 were downloaded from

sequencing read archive (SRA) and velocyto was run on these data using mouse genome mm10 with repeatmask for mm10 (*19*, *20*). Matrix data and velocyto output were loaded into one adata object in scanpy (*21*). QC filtering was already perfomed on the matrix files and therefore, only cells with less than 500 genes detected and genes found in less than 20 cells were filtered out. Counts were normalized to log(count+1) using the natural logarithm. An UMAP was created with 50 PCA dimensions. Cell clusters were named based on comparison to Haber et als gene sets for cell types (*1*). Tuft cells where then selected and subgrouped into two clusters using the leiden algorithm with a resolution of 0.09. RNA velocity was calculated and plotted with scVelo (*22*).

Supplementary Tables

Supplementary file 1 - GSEA gene sets

All gene sets used for GSEA. See file *Supplementary_file_1_gsea.xlsx*.

Supplementary file 2 - Goblet cell gene signatures

Goblet cell genes from plate based scRNAseq compared to genes up-regulated in 24 hour treatment with IL-13 and IL-22. See file *Supplementary_file_3_goblet_cell_signatures.xlsx*

References

1. A. L. Haber, M. Biton, N. Rogel, R. H. Herbst, K. Shekhar, C. Smillie, G. Burgin, T. M. Delorey, M. R. Howitt, Y. Katz, I. Tirosh, S. Beyaz, D. Dionne, M. Zhang, R. Raychowdhury, W. S. Garrett, O. Rozenblatt-Rosen, H. N. Shi, O. Yilmaz, R. J. Xavier, A. Regev, en, *Nature* **551**, 333–339, ISSN: 0028-0836, 1476-4687 (Nov. 2017).

2. M. Biton, A. L. Haber, N. Rogel, G. Burgin, S. Beyaz, A. Schnell, O. Ashenberg, C.-W. Su, C. Smillie, K. Shekhar, Z. Chen, C. Wu, J. Ordovas-Montanes, D. Alvarez, R. H. Herbst, M. Zhang, I. Tirosh, D. Dionne, L. T. Nguyen, M. E. Xifaras, A. K. Shalek, U. H. von Andrian, D. B. Graham, O. Rozenblatt-Rosen, H. N. Shi, V. Kuchroo, O. H. Yilmaz, A. Regev, R. J. Xavier, en, *Cell* **175**, 1307–1320.e22, ISSN: 00928674 (Nov. 2018).

3. M. Camberis, G. Le Gros, J. Urban, eng, *Current Protocols in Immunology* **Chapter 19**, Unit 19.12, ISSN: 1934-368X (Aug. 2003).

4. T. Sato, H. Clevers, en, in *Epithelial Cell Culture Protocols: Second Edition*, ed. by S. H. Randell, M. L. Fulcher (Humana Press, Totowa, NJ, 2013), pp. 319–328, ISBN: 978-1-62703-125-7.

5. G. Bradski, *The OpenCV Library*, Library Catalog: www.drdobbs.com, 2000, (`http://www.drdobbs.com/open-source/the-opencv-library/184404319`).

6. M. Abadi, A. Agarwal, P. Barham, E. Brevdo, Z. Chen, C. Citro, G. S. Corrado, A. Davis, J. Dean, M. Devin, S. Ghemawat, I. Goodfellow, A. Harp, G. Irving, M. Isard, Y. Jia, R. Jozefowicz, L. Kaiser, M. Kudlur, J. Levenberg, D. Mane, R. Monga, S. Moore, D. Murray, C. Olah, M. Schuster, J. Shlens, B. Steiner, I. Sutskever, K. Talwar, P. Tucker, V. Vanhoucke, V. Vasudevan, F. Viegas, O. Vinyals, P. Warden, M. Wattenberg, M. Wicke, Y. Yu, X. Zheng, *arXiv* (2015).

7. *Keras*, 2015, (`https://keras.io/`).

8. H. Wickham, *ggplot2: Elegant Graphics for Data Analysis* (Springer-Verlag New York, 2016), ISBN: 978-3-319-24277-4.

9. A. Dobin, C. A. Davis, F. Schlesinger, J. Drenkow, C. Zaleski, S. Jha, P. Batut, M. Chaisson, T. R. Gingeras, en, *Bioinformatics* **29**, 15–21, ISSN: 1367-4803 (Jan. 2013).

10. A. Frankish, M. Diekhans, A.-M. Ferreira, R. Johnson, I. Jungreis, J. Loveland, J. M. Mudge, C. Sisu, J. Wright, J. Armstrong, I. Barnes, A. Berry, A. Bignell, S. Carbonell Sala, J. Chrast, F. Cunningham, T. Di Domenico, S. Donaldson, I. T. Fiddes, C. García Girón, J. M. Gonzalez,

T. Grego, M. Hardy, T. Hourlier, T. Hunt, O. G. Izuogu, J. Lagarde, F. J. Martin, L. Martínez, S. Mohanan, P. Muir, F. C. P. Navarro, A. Parker, B. Pei, F. Pozo, M. Ruffier, B. M. Schmitt, E. Stapleton, M.-M. Suner, I. Sycheva, B. Uszczynska-Ratajczak, J. Xu, A. Yates, D. Zerbino, Y. Zhang, B. Aken, J. S. Choudhary, M. Gerstein, R. Guigó, T. J. P. Hubbard, M. Kellis, B. Paten, A. Reymond, M. L. Tress, P. Flicek, en, *Nucleic Acids Research* **47**, D766–D773, ISSN: 0305-1048 (Jan. 2019).

11. Y. Liao, G. K. Smyth, W. Shi, en, *Bioinformatics* **30**, 923–930, ISSN: 1367-4803 (Apr. 2014).

12. M. I. Love, W. Huber, S. Anders, *Genome Biology* **15**, 550, ISSN: 1474-760X (Dec. 2014).

13. K. Blighe, *Publication-ready volcano plots with enhanced colouring and labeling: kevinblighe/EnhancedVol* original-date: 2018-02-16T13:36:36Z, Feb. 2019, (https://github.com/kevinblighe/EnhancedVolcano).

14. F. Pedregosa, G. Varoquaux, A. Gramfort, V. Michel, B. Thirion, O. Grisel, M. Blondel, P. Prettenhofer, R. Weiss, V. Dubourg, J. Vanderplas, A. Passos, D. Cournapeau, en, *MACHINE LEARNING IN PYTHON*, 6 (2011).

15. G. Yu, L.-G. Wang, Y. Han, Q.-Y. He, *OMICS: A Journal of Integrative Biology* **16**, 284–287 (Mar. 2012).

16. R. Kolde, *pheatmap: Pretty Heatmaps*, 2019, (https://CRAN.R-project.org/package=pheatmap).

17. J. Larsson, *eulerr: Area-Proportional Euler and Venn Diagrams with Ellipses*, 2019, (https://cran.r-project.org/package=eulerr).

18. M. M. Alonso, H. Lindholm, S. Iqbal, P. Vornewald, S. Hoel, M. Altelaar, P. Katajisto, A. Arroyo, M. Oudhoff, *bioRxiv* (2020).

19. G. La Manno, R. Soldatov, A. Zeisel, E. Braun, H. Hochgerner, V. Petukhov, K. Lidschreiber, M. E. Kastriti, P. Lönnerberg, A. Furlan, J. Fan, L. E. Borm, Z. Liu, D. van Bruggen, J. Guo, X. He, R. Barker, E. Sundström, G. Castelo-Branco, P. Cramer, I. Adameyko, S. Linnarsson, P. V. Kharchenko, en, *Nature* **560**, Bandiera_abtest: a Cg_type: Nature Research Journals

Number: 7719 Primary_atype: Research Publisher: Nature Publishing Group Subject_term: Differentiation;Genome informatics Subject_term_id: differentiation;genome-informatics, 494–498, ISSN: 1476-4687 (Aug. 2018).

20. W. Bao, K. K. Kojima, O. Kohany, *Mobile DNA* **6**, 11, ISSN: 1759-8753 (June 2015).

21. F. A. Wolf, P. Angerer, F. J. Theis, *Genome Biology* **19**, 15, ISSN: 1474-760X (Feb. 2018).

22. V. Bergen, M. Lange, S. Peidli, F. A. Wolf, F. J. Theis, en, *Nature Biotechnology* **38**, 1408–1414, ISSN: 1087-0156, 1546-1696 (Dec. 2020).
